# Cell type-specific termination of transcription by transposable element sequences

**DOI:** 10.1186/1759-8753-3-15

**Published:** 2012-09-30

**Authors:** Andrew B Conley, I King Jordan

**Affiliations:** 1 School of Biology, Georgia Institute of Technology, 310 Ferst Drive, Atlanta, GA 30332, USA; 2 PanAmerican Bioinformatics Institute, Santa Marta, Magdalena, Colombia

**Keywords:** Polyadenylation, Transcription termination, Orientation bias, Gene regulation

## Abstract

**Background:**

Transposable elements (TEs) encode sequences necessary for their own transposition, including signals required for the termination of transcription. TE sequences within the introns of human genes show an antisense orientation bias, which has been proposed to reflect selection against TE sequences in the sense orientation owing to their ability to terminate the transcription of host gene transcripts. While there is evidence in support of this model for some elements, the extent to which TE sequences actually terminate transcription of human gene across the genome remains an open question.

**Results:**

Using high-throughput sequencing data, we have characterized over 9,000 distinct TE-derived sequences that provide transcription termination sites for 5,747 human genes across eight different cell types. Rarefaction curve analysis suggests that there may be twice as many TE-derived termination sites (TE-TTS) genome-wide among all human cell types. The local chromatin environment for these TE-TTS is similar to that seen for 3^′^ UTR canonical TTS and distinct from the chromatin environment of other intragenic TE sequences. However, those TE-TTS located within the introns of human genes were found to be far more cell type-specific than the canonical TTS. TE-TTS were much more likely to be found in the sense orientation than other intragenic TE sequences of the same TE family and TE-TTS in the sense orientation terminate transcription more efficiently than those found in the antisense orientation. Alu sequences were found to provide a large number of relatively weak TTS, whereas LTR elements provided a smaller number of much stronger TTS.

**Conclusions:**

TE sequences provide numerous termination sites to human genes, and TE-derived TTS are particularly cell type-specific. Thus, TE sequences provide a powerful mechanism for the diversification of transcriptional profiles between cell types and among evolutionary lineages, since most TE-TTS are evolutionarily young. The extent of transcription termination by TEs seen here, along with the preference for sense-oriented TE insertions to provide TTS, is consistent with the observed antisense orientation bias of human TEs.

## Background

For any individual human, different kinds of somatic cells contain the same genome sequence, but are obviously functionally distinct. Thus, cell type-specific regulation of the genome, rather than the sequence itself, defines the characteristics of a cell type. The importance of cell type-specific activity of promoters in the functional differentiation cell types has long been appreciated; however, the role of cell type-specific termination of transcription in this process has not been as well studied. Nevertheless, recent studies have begun to show that variation in transcription termination is important for cell-type specification [1-3] and have piqued an interest in this largely unexplored phenomenon.

There are numerous transposable element (TE)-derived sequences in the human genome [4], comprising more than two-thirds of the total sequence [5], and many of these TEs are located within the introns of human genes. TEs contain their own regulatory sequences, including specific signals that lead to the termination of transcripts initiated from element promoters. Human endogenous retroviral elements (HERVs), for example, have polyadenylation signals in their long terminal repeat (LTR) regions that terminate transcription [6]. Thus, numerous TE sequences located within, or nearby, human gene sequences may contribute substantially to the termination of gene transcription via the provisioning of termination signals.

There are several known examples whereby TE sequences located within, or nearby, human genes have been shown to terminate transcription of genic mRNAs. An early study of HERVs provided the first direct evidence that TE-derived sequences can terminate the transcription of non-TE human mRNAs and further suggested that different subfamilies of these elements may serve to terminate transcription in a cell type-specific manner [7]. Later, the same family of ERVs was demonstrated to terminate transcription of a novel alternatively spliced version of the human NAAA gene [8]. There is also experimental evidence showing that L1 (LINE) retrotransposon sequences can terminate the transcription of human genes, and in this same study the intronic content of L1 sequences in human genes was found to be negatively correlated with their expression levels [9]. A later study showed a similar trend whereby the presence of polymorphic L1 insertions in human genes was correlated with a decrease in their expression in a tissue-specific manner [10].

Despite the evidence cited above indicating that TE sequences can terminate transcription of human genes in a cell type-specific manner in some cases, the extent of this phenomenon and its overall effect on cell type-specific expression have not been fully explored. A pair of recent genome-scale surveys of transcription termination by TEs revealed ~3,000 cases of human transcripts that terminate with TEs [11,12], suggesting that the phenomenon may be widespread. These studies, while intriguing, relied on relatively low throughput transcriptomic technologies and did not address the cell type specificity of TE transcription termination. Thus, the full extent of TE transcription termination within the human genome, and equally as important the cell type specificity of this phenomenon, remains unknown.

Here, we deeply interrogated the contribution of TE sequences to human gene transcription termination via the integrated analysis of high-throughput transcriptomic data and TE gene annotations. Since TE sequences have been shown to contribute disproportionately to cell type-specific regulation [13], we also evaluated the extent to which that transcription termination of human genes by TEs is cell type-specific. To do this, we characterized the space of transcription termination sites (TTS) derived from TE insertions in eight different ENCODE cell types. For these TE-TTS, we characterized the contributions from different TE families, as well as their relative insertion orientations. We found 9,287 TE-derived sequences that terminate the transcription of 5,747 human genes. Our results also show that TEs terminate transcript much more efficiently when inserted in the sense orientation relative to gene transcription and thus lend credence to the previously articulated notion that TE orientation biases result from selection against TE termination of gene transcription. We also show that TE termination of gene transcription is highly cell type-specific and thus may contribute to the specialization of cellular function through differential gene regulation.

## Results and discussion

### Characterization of transposable element-derived termination sites

We characterized TE sequences that provide transcription termination sites (TE-TTS) to human genes using Paired-End diTag (PET) data. PET is a technique for the high-throughput characterization of the 5^′^ and 3^′^ ends of mature full-length mRNAs [14], which allows for deep annotation of paired transcription start (TSS) and termination sites (TTS), including the discovery of many novel alternative sites. TE-TTS were characterized by co-locating TE sequences with 3^′^ PET tag clusters that are paired with 5^′^ PET tag clusters mapped to known human gene promoters (see Methods, Additional file 1: Table S1). Using PET data from eight different ENCODE cell types (GM12878, H1HESC, HeLaS3, HepG2, HUVEC, K562, NHEK and Prostate) [15,16], we discovered 98,632 total TTS, 9,287 of which are derived from TE sequences. Thus, 9.4% of human gene TTS are provided by TE-derived sequences, and 28% of human gene loci have at least one TE-TTS.

The breakdown of TSS contributed by different TE families, and the locations of these TE-TTS within human gene loci are shown in Table 1. While many TE-TTS correspond to the 3^′^UTRs and canonical TTS of human genes (21%), the majority of TE-TTS represents alternative TTS found within gene boundaries (70%) and yield creating truncated transcripts. A small minority of these alternate TE-TTS (8%) is found within upstream of coding sequences, representing messages that are severely truncated or aborted albeit in a site-specific and reproducible manner. In addition to this, there is an enrichment of TE-TTS toward the 5^′^-end of genes (Additional file 1: Figure S4). Transcripts using these TE-TTS would likewise generate highly truncated transcripts. TE sequences also provide TTS downstream of the canonical TTS of human genes (8%), providing longer alternative transcripts. Overall, 87% of the total TE-TTS locations correspond to alternative TTS compared to 81% for non-TE-TTS, indicating that TE sequences are utilized as alternative terminators at a slightly higher frequency than non-TE sequences. Interestingly, with the exception of Alu elements, TE-TTS are distributed along the length of the element consensus sequence and thus represent cryptic termination sites as seen previously for L1 elements [9].

**Table 1:** Locations of human gene transcription termination sites (TTS) characterized using PET data

**TTS Location**^**a**^	**TE Family**^**b**^									
	**Non-TE**	**All TE**	**Alu**	**ERV**	**hAT**	**L1**	**L2**	**MaLR**	**MIR**	**TcMar**
**5′-UTR**	3,677	696	347 (49%)	52 (7%)	34 (4%)	113 (16%)	57 (8%)	17 (2%)	54 (7%)	22 (3%)
**Internal**	46,716	6,014	2,955 (55%)	162 (3%)	332 (6%)	842 (15%)	371 (6%)	110 (2%)	377 (7%)	158 (2%)
**3′-UTR**	15,491	867	267 (37%)	25 (3%)	69 (9%)	120 (16%)	60 (8%)	29 (4%)	101 (14%)	43 (6%)
**Annotated**^**c**^	16,031	1,310	291 (25%)	109 (9%)	102 (9%)	229 (20%)	123 (10%)	67 (5%)	150 (13%)	59 (5%)
**Downstream**^**d**^	2,806	804	222 (33%)	70 (10%)	51 (7%)	141 (21%)	49 (7%)	45 (6%)	53 (7%)	33 (4%)
**Sum**	84,721	8,511	4,082 (47%)	418 (4%)	588 (6%)	1,445 (16%)	660 (7%)	268 (3%)	735 (8%)	315 (3%)

^a^The locations of TTS characterized using PET data from eight ENCODE cell types were characterized relative to known human gene models Percentages shown are the percent of TTS in a location derived from a TE family.

^b^TTS genic locations are shown for non TE-TTS and for the top eight TTS contributing TE families.

^c^TTS located within 250 bp of canonical TTS.

^d^TTS located up to 5 kb downstream of previously canonical TTS.

**Figure 1:**
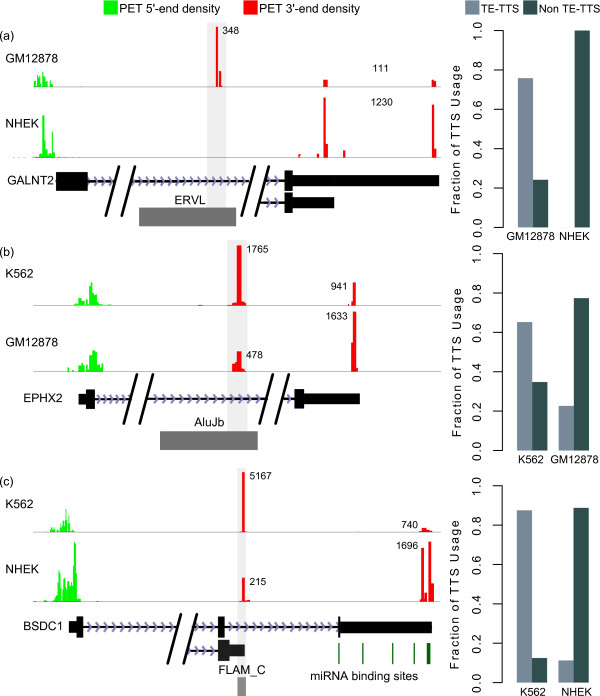
**TE insertions terminate transcription in a cell type-specific manner.** Clusters of linked paired-end ditag (PET) sequences that indicate the locations of the 5 (green) and 3 (red) ends of full-length transcripts expressed in different cell types are shown above gene models indicating the locations of exons, introns and TEs that terminate transcription. For each example, the cell type-specific fractions of TTS usage for TE-TTS and non-TE-TTS are shown. (**a**) An ERVL insertion within the first intron of the GALNT2 gene terminates the majority of transcripts in the GM12878 cell type, with a small number terminating in the two canonical TTS. No transcripts terminate within the ERVL in the NHEK cell type. (**b**) An AluJb insertion within the seventh intron of the EPHX2 gene terminates the majority of transcripts in the K562 cell type, while the majority of transcripts read through this sequence in the GM12878 cell type. (**c**) Termination of transcription within a FLAM_C insertion in the BSDC1 gene results in a shortened 3′UTR and altered C-terminal coding region. The FLAM_C-derived TTS is utilized extensively in the K562 cell type, while the majority of transcripts read through this sequence in the NHEK cell type.

Several examples of human genes with TE-TTS are show in Figure 1. Transcription initiated from the GALNT2 promoter can terminate within an ERVL insertion in the first intron of the locus or in two canonical TTS in the 3′ UTR (Figure 1a). TE-derived termination of GALNT2 occurs in a cell type-specific manner; most GALNT2 transcripts (78%) utilize the ERVL-derived TTS in the GM12878 cell type, whereas virtually all GALNT2 transcripts read through the ERVL insertion in the NHEK cell type and instead utilize the two canonical TTS (2 × 2 *χ*^2^ = 1,169, *P* ≈ 0). The transcript resulting from utilization of the ERVL-derived TTS is severely truncated and therefore highly unlikely to produce a functional protein. Thus, while this gene is transcribed at high levels in both cell types, the ERVL-derived terminator serves to effectively reduce GALNT2 expression in GM12878 compared to NHEK. Similarly, EPHX2 transcription can terminate within an AluJb insertion in the sixth intron, resulting in a truncated transcript (Figure 1b). This termination is also cell type-specific, with the majority of transcripts (66%) utilizing the AluJb-derived TTS in the K562 cell type and a minority (24%) GM12878 (2 × 2 *χ*^2^ = 862, *P* ≈ 0).

Though many alternative TE-derived TTS occur within an intron of a coding locus as seen for GALNT2 and EPHX2, some TE-TTS may leave ORF intact or nearly so. For example, a TTS derived from a FLAM_C TE sequence in the BSDC1 gene is found at an alternative upstream position in the terminal intron (Figure 1c). Indeed, a human mRNA from GenBank contains this TTS and suggests an alternative C-terminal coding sequence. The canonical BSDC1 TTS is found several kb downstream of the TE-TTS, and the resulting 3^′^UTR contains ten miRNA binding sites that could be used to degrade the mRNA or reduce its translation. Thus, utilization of the FLAM_C-derived TTS, which would generate a transcript with a nearly full-length ORF but a drastically shortened 3^′^UTR lacking miRNA binding sites, could effectively increase the expression of BSDC1 by evading post-transcriptional regulation via miRNA binding. As is the case for the GALNT2 and EPHX2 genes, the utilization of this TE-TTS is cell type-specific, with the majority of transcripts in K562 utilizing the FLAM_C-derived TTS and the majority reading through the TE-TTS in NHEK cells (2 × 2 *χ*^2^ = 3,907, *P* ≈ 0). The contribution of TE sequences to alternative transcription termination is further explored later in the manuscript.

**Figure 2:**
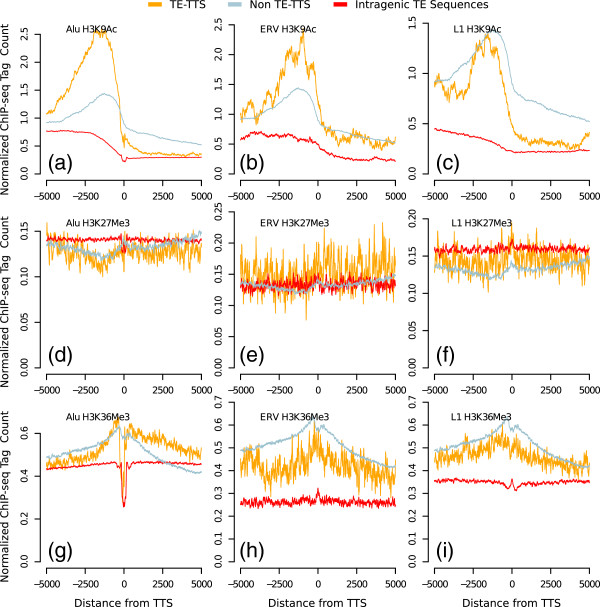
**The chromatin environment of TE-TTS is similar to that of non-TE-TTS and distinct from intragenic TE sequences that do not terminate transcription.** The locations of TTS and the ChIP-seq tag counts corresponding to H3K9Ac (**a**-**c**), H3K27Me3 (**d**-**f**) and H3K36Me3 (**g**-**i**) are shown for the K562 cell type. Enrichment curves, showing the average normalized numbers of ChIP-seq tags in ten base-pair windows ±5 kb of TE-TTS (orange), non-TE-TTS (gray) and intragenic TE sequences that do not show a TTS (red), are shown for three TE families, Alu (**a**,**d**,**g**), ERV (**b**,**e**,**h**) and L1 (,**c**,**f**,**i**).

In an effort to further characterize the TE-TTS discovered here, we used ENCODE ChIP-seq data for the locations of histone modifications [15-17] to evaluate their local chromatin environment. We found that the histone modification signatures of TE-TTS are generally similar to those of non TE-TTS and distinct from intragenic TE insertions that do not provide a TTS. Different histone modifications showed distinct patterns of enrichment near TTS, and we show representative examples of TTS histone modification signatures for an active transcriptional mark (H3K9Ac), a mark of transcriptional elongation and gene boundaries (H3K36Me), and a repressive mark (H3K27Me3) in the K562 cell type. H3K9Ac shows a marked peak of enrichment upstream of both TE-TTS and non TE-TTS, and then the levels fall off precipitously after the TSS (Figure 2a-c). H3K27Me3 shows a slight increase downstream of the TTS for non-TE-TTS; however, the enrichment level was generally very low (~ 0.1 tags per million mapped). This downstream increase in H3K27Me3 was not seen for the TE-TTS (Figure 2d-f), though this could be due to the comparatively low number of TE-TTS compared to non TE-TTS together with the relatively low number of H3K27Me3 marks seen within actively transcribed genes. The H3K36Me3 modification shows a more symmetrical distribution around TTS with peaks for both TE-TTS and non-TE-TTS compared to intragenic TEs that do not show TSS-related peaks (Figure 2g-i). Qualitatively similar results were seen in the GM12878 and NHEK cell types (Additional file 1: Figures S2-S3). Overall, the similar local chromatin environments seen for TE-TTS and non-TE-TTS suggest that the TE-TTS characterized represent *bona fide* terminators as opposed to transcriptional noise. A similar enrichment of these histone modifications was seen previously using ChIP-seq data from CD4^+^ T-cells [18].

### TE transcriptional termination and insertion orientation bias

The vast majority of TE sequences within human genes are found in the antisense orientation relative to the direction of transcription of the gene [19]. The genic orientation bias of human TEs is thought to reflect differential selective elimination of sense TE insertions over time rather than a preference in the introduction of antisense insertions at the moment of transposition. The ability of TEs to cause premature termination of gene transcripts, thereby reducing levels of transcription, has been proposed as a mechanism to explain the selective elimination of sense oriented L1 sequences from human gene loci [9]. In order to investigate the role of TE-TTS in the selection against sense-oriented TE insertions genome-wide, we compared the insertion orientations of intragenic TEs that do not provide TTS versus the orientations of TE-TTS for the eight largest families of human TEs (Alu, ERV, hAT, L1, L2, MaLR, MIR and TcMar).

**Figure 3:**
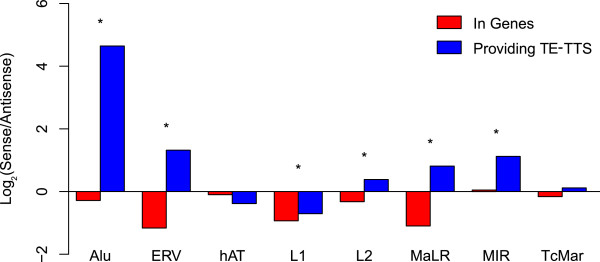
**TE sequences providing transcription termination sites show a strong sense bias.** For each TE family, the sense/anti-sense ratio was determined for all intragenic insertion (red) and only for those TEs that provide a TE-TTS (blue). For each TE family, statistical significance levels for the differences in the sense/anti-sense ratios (*P < 0.005) were determined using a chi-squared distribution with *df* = 1.

Seven out of eight TE families show the expected antisense orientation bias for intragenic TE insertions for which there is no evidence of TTS activity (Figure 3). In other words, since these antisense TE insertions do not terminate transcription, their presence within human genes is tolerated by selection. The LTR element families, the ERVs and MaLRs, show the strongest antisense orientation bias, with intragenic insertions being found in the antisense orientation twice as often as the sense orientation. Conversely, Alu insertions show a much weaker antisense orientation bias. The relatively stronger bias seen for LTR element insertions suggests the possibility that there is stronger selection against sense LTR insertions and that such sense LTR element insertions may be more deleterious. This point is explored in more detail later in the manuscript.

For those genic TE sequences that provide a TTS, the majority of TE families show a significant enrichment of insertions in the sense orientation versus the other insertions. Alus have one of the weaker antisense orientation biases for genic elements, but Alu-derived TTS show far and away the strongest sense bias; an Alu insertion providing a TTS is approximately 20× more likely to be in the sense orientation than the antisense orientation. While LTR element genic insertions show the strongest overall antisense bias, insertions providing a TTS are also much more likely to be in the sense orientation; an LTR element providing a TTS is 4× more likely to be found in the sense orientation than the average genic LTR element insertion. The strong sense orientation enrichment seen for TE-TTS indicates that genic TEs oriented in the same direction as transcription are much more effective transcription terminators, consistent with the notion that sense-oriented TE insertions are selected against owing to their disruptive effects on gene expression.

The only exception to this pattern is seen for the relatively ancient family of MIR TEs. MIRs have previously been implicated in providing gene regulatory sequences in a number of studies [20,21], and the MIR sequences that remain intact and recognizable in the human genome are likely to have been conserved by purifying selection [22]. Thus, the lack of orientation bias for MIRs, irrespective of their status as TTS, may reflect their general utility as gene regulators rather than an ephemeral presence as neutral sequences that will be eventually lost by mutational decay.

### Contributions of Alus to transcriptional termination

**Figure 4:**
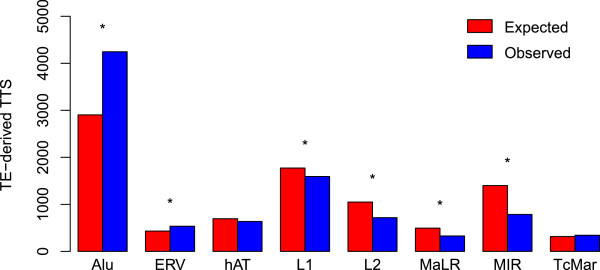
**Alu family sequences provide a greater than expected number of TTS.** Expected (red) versus observed (blue) counts of TTS derived from different TE families. Expected counts of TTS derived from each TE family were calculated based on the fraction of intragenic sequences. For each TE family, statistical significance levels for the differences between the expected versus observed counts (*P <10^-5^) were determined using a chi-squared distribution with *df* = 1.

Given the diversity of TE insertions found in and around human genes, we sought to characterize the relative TE-TTS contributions of the eight largest families of human TEs (Alu, ERV, hAT, L1, L2, MaLR, MIR and TcMar). To do this, we compared the observed numbers of TE-TTS for the different families to the expected numbers based on their genic frequencies (Figure 4). While L1s contribute the most TE sequence genome-wide, Alus are the most abundant genic TE family (31% of all genic TE insertions) (RepeatMasker). Thus, Alu insertions would be expected to provide a large number of TE-derived TTS. However, previous studies have characterized ~400 Alu insertions providing TTS, a substantially smaller than expected fraction [11,12]. In contrast to these findings, we found that Alu-TTS were more abundant than TTS derived from other TE families, providing 43% percent (4,551) of all TE-TTS, far more than would be expected based on the frequency of Alu genic insertions. Other TE families generally contributed fewer TTS than expected based on their genic frequencies, with MIR-derived TTS being far less common than expected; ERV was the only other TE family to provide significantly more TTS than expected.

**Figure 5:**
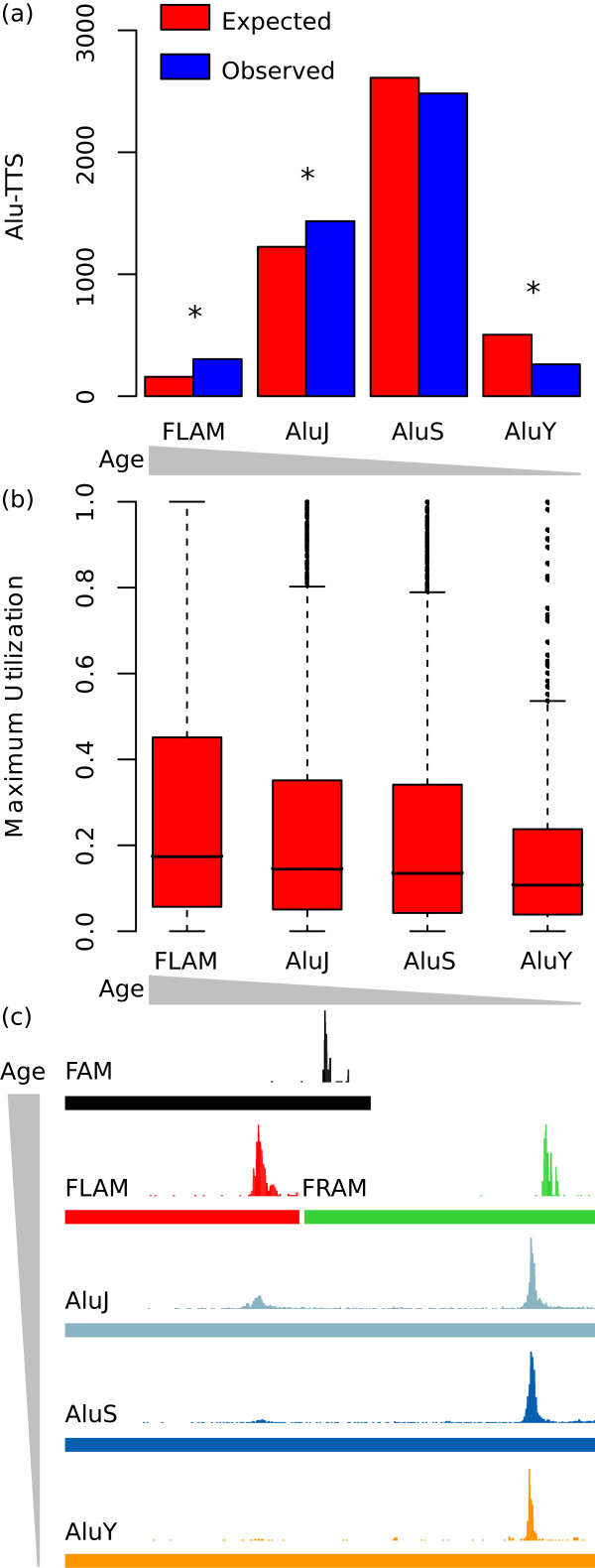
**Alu-TTS are not randomly distributed in Alu insertions and older Alu families are overrepresented.** (**a**) Expected (red) versus observed (blue) counts of Alu-TTS are shown for individual subfamilies of different ages (older-left to younger-right). Expected counts of TTS derived from each subfamily were calculated based on the fraction of intragenic sequences. For each Alu subfamily, statistical significance levels for the differences between the expected versus observed counts (*P < 10^-4^) were determined using a chi-squared distribution with *df* = 1. (**b**) Distributions of maximum utilization values (see Methods) for Alu-TTS are shown for individual subfamilies of different ages (older-left to younger-right). (**c**) For Alu-TTS provided by elements of different subfamilies, the position of each TTS within the subfamily consensus sequence was determined, and the density of all TTS along the length of each subfamily consensus sequence is indicated by the height of the peaks.

The overabundance of Alu-TTS could be attributed to their functional utility as expression regulators, or it could simply reflect the fact that Alu-TTS are not as disruptive and therefore more tolerated by selection. Consistent with the latter neutral scenario, the overabundance of Alu-TTS may reflect their relatively young age, suggesting that there has not been adequate time for their removal from the genome. To evaluate these possibilities, we evaluated the TTS contributions of Alu subfamilies of different ages (FLAM, AluJ, AluS and AluY). Relatively older Alu subfamilies (FLAM and AluJ) contribute more TTS than expected, whereas the younger subfamilies (AluS and AluY) contribute fewer than expected (Figure 5a). For instance, even though FLAM elements are found in less than half the genic frequency of AluY insertions, they contribute more TTS to human genes. These observations argue against the neutral explanation for the abundance of Alu-TTS. It is formally possible that the overrepresentation of older Alu subfamilies among TE-TTS is an artifact caused by ambiguous mapping of the short sequence tags used in PET. Such ambiguous mapping could cause an apparent underrepresentation of younger families, members of which are more similar in sequence (i.e., more redundant) and would thus be less mappable. To control for this possibility, we repeated the same analysis using PET tags of different sizes (16 bp or 25 bp). The overrepresentation of older Alu subfamilies among TE-TTS is the same for both sets of data (Additional file 1: Figure S5), indicating that this result is not an artifact of ambiguous mapping of short PET tags.

To further explore the contributions of the different Alu subfamilies, we evaluated the strength of utilization for TTS derived from the different subfamilies. The strength of utilization for any TTS is measured as the relative frequency with which it terminates transcription versus the frequency that it is read through (see Methods). Consistent with what is seen for the relative levels of TTS donation by the different Alu subfamilies, older families show higher levels of TTS utilization than do younger families (Figure 5b), suggesting the possibility that many of these Alu-TTS are preserved via selection by virtue of their functional utility for the host gene.

In light of the exceptional ability of Alus to provide TTS to human genes, we explored the specific sequence context by which these elements terminate transcription. To do this, we mapped the locations of Alu-derived TTS to their positions in the Alu subfamily consensus sequences [23]. Previously, when a few hundred Alu-TTS were considered as an ensemble, they were found to terminate human gene transcription non-randomly at two specific locations along their sequence [11,12]. For this study, by considering thousands of Alu-TTS among individual Alu subfamilies of different relative ages, we were able to tease apart this apparently bimodal pattern of termination and discern its origins. The modern Alu element is a dimeric sequence composed of two related precursor sequences: a Free Left Alu Monomer (FLAM) and Free Right Alu Monomer (FRAM) [24,25]. These sequences themselves descended from the Fossil Alu Monomer (FAM), which in turn descended from a 7SL RNA [25]. Elements from all three families of Alu precursors terminate transcription at single site near their 3^′^-end (Figure 5c). However, when the FLAM and FRAM monomers are considered with respect to their homologous locations in the descendent Alu dimer sequences, these individual termination sites yield a pair a corresponding termination sites: one internal termination site corresponding to the FLAM 3^′^ site and a 3^′^ termination site corresponding to the FRAM 3′site. In modern Alus, the 3^′^ termination site predominates over the internal site, and the use of the internal site markedly decreases among younger element sequences (Figure 5c). The attenuation in the strength of this TTS donating site from the internal region of the element may reflect the need of the elements themselves to produce full-length transcripts in order to be transposed. In this case, selection against the internal TTS site would be at the level of the element as opposed to at the level of the host. Thus, the steady migration over time of the Alu-TTS donating site to the 3^′^ end of the element reflects a complex dynamic between inter-element selection and the effects that the elements can in turn exert on their host genome.

It should be noted that the TTS-enriched positions for Alu subfamilies seen in Figure 5 are upstream to oligo-A sequences found in the elements. Since the PET technique utilizes poly-dT primers for the generation of cDNAs, apparent TTS associated with such oligo-A sequences could represent artifacts of internal priming on mRNA sequences. While the PET technique does include a biotin enrichment step that is designed to eliminate such non full-length cDNAs generated from internal priming [14], it is formally possible that some experimental artifacts remain after this step. We implemented a series of controls in order to ensure that the Alu-TTS observed here are not likely to represent experimental artifacts from internal priming. Methodological details of these controls and the results can be found in Additional file 1: Tables S3-S5, Figures S6-S9. Overall, Alu-TTS characteristics are not consistent with internal priming PET artifacts, and the chromatin environment of Alu-TTS closely resembles the chromatin environment of non TE-TTS and differs markedly from the chromatin environment of genic Alus.

### Relative levels of utilization for TE-derived TTS

**Figure 6:**
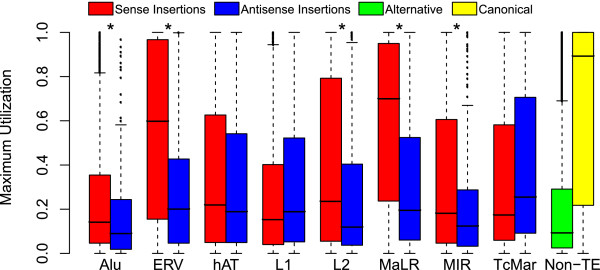
**LTR-TTS are more strongly utilized than TE-TTS provided by other families.** Distributions of maximum utilization values (see Methods) are shown for TE-TTS from different families along with alternative (green) and canonical annotated (yellow) TTS. TE-TTS maximum utilization values are shown separately for sense (red) and antisense (blue) insertions. Statistical significance levels for the differences between the maximum utilization insertion orientations for each TE family (*P < 0.005) were determined using a Wilcoxon rank-sum test.

The eight human TE families evaluated here have diverse evolutionary origins, methods of transposition and sequence composition. Given these differences, it would be reasonable to expect that TTS derived from the different TE families would behave differently. To assess whether this is the case, we compared the strength of utilization (see Methods) for TTS derived from members of the different TE families along with the utilization levels seen for non TE-TTS. Individual TTS derived from Alu insertions, while being by far the most abundant TE-derived TTS in the genome (Table 1 and Figure 4), are utilized far less frequently than TE-TTS derived from other families or non-TE-TTS (Figure 6). This finding is in accordance with the weak transcription termination previously seen for Alus [26]. On the opposite extreme, TTS derived from sense LTR element insertions, including both the ERV and MaLR families, are utilized significantly more frequently than TTS from any other TE family or alternative non TE-TTS. Indeed, 25% of TTS derived from LTR element insertions have a maximum utilization of over 90% in at least one of the ENCODE cell types. The only group of TTS that shows higher maximum utilization is the group of previously annotated canonical non TE-TTS. The large differences in the relative strengths of Alu and LTR element-derived TTS may explain the differences seen in the orientation biases between these families (Figure 3). The idea is that Alu insertions provide weaker TTS and thus may be more tolerated in the sense orientation, while ERV and MaLR insertions provide strong TTS and thus sense-oriented LTRs are strongly selected against.

The L1 family is curious, being the only TE family to show a strong antisense bias for those insertions providing a TTS (Figure 3), yet at the same time showing no difference in TTS strength of utilization between sense and antisense insertions (Figure 6). Han *et al.* showed that L1 insertions are capable of terminating transcription in either the sense or antisense orientation, with several polyadenylation signals occurring in the antisense orientation [9]. The same study also showed that L1 insertions can cause transcriptional disruption when in the sense orientation, independent of polyadenylation. As the PET technique requires that transcripts be polyadenylated, the data used here cannot take into account non-polyadenylated transcriptional disruption by L1s. Therefore, the anomalous L1 patterns observed here with respect to both TTS orientation bias and strength of utilization may reflect the relative usage of polyadenylation in L1-TTS from the different strands.

In light of the results on the orientation bias of TE-TTS (Figure 3), we also compared the strength of utilization for TE-TTS found in sense versus antisense orientations relative to the direction of transcription. Five out of eight of the TE families (Alu, ERV, L2, MaLR and MIR) showed a significant difference (*P <*0.01, Wilcoxon rank-sum test) in TTS strength of utilization depending on the orientation of the insertion. In all five of these families, TTS derived from sense insertions are more likely to be utilized than those derived from antisense insertions (Figure 6). These results are consistent with the findings from the overall TE orientation bias in human genes, suggesting that selection acts to remove TE-derived terminators that disrupt gene expression.

### Cell type-specific regulatory potential of TE-TTS

**Figure 7:**
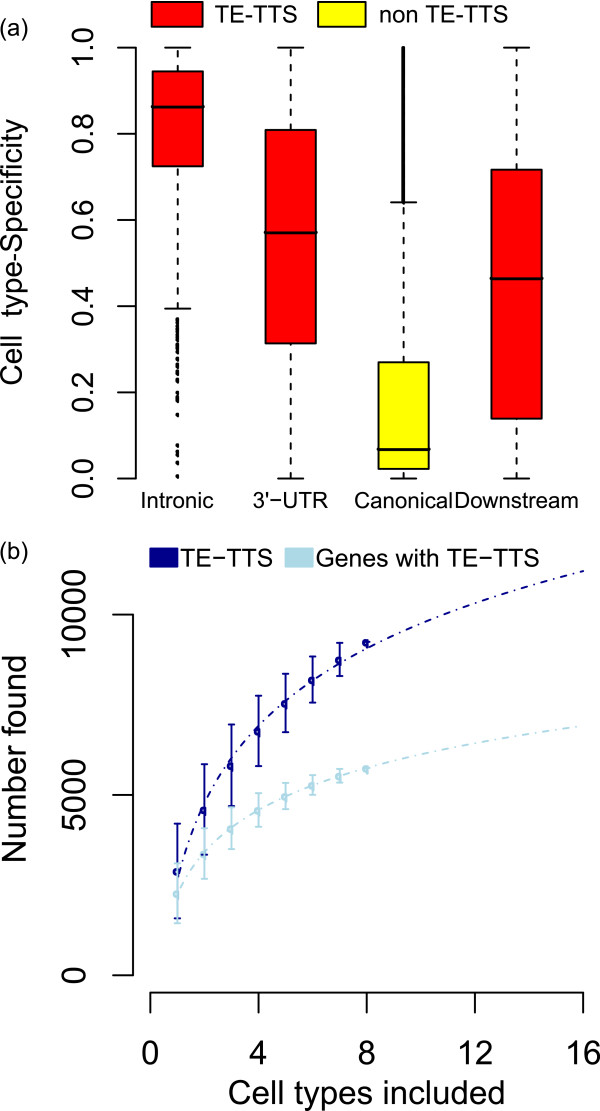
**TE-TTS terminate transcription in a cell type-specific manner.** (**a**) Cell type specificity value distributions are shown separately for TE-TTS (red) located within introns, 3^′^ UTRs and downstream of annotated TTS. Cell-type specificity values are also shown for canonical annotated TTS (yellow). (**b**) Rarefaction curves showing the average numbers (±SD) of TE-TTS (dark blue) and genes with at least one TE-TSS (light blue) detected when all possible combinations of 1–8 cell types are used. Observed curves are fitted with a logarithmic trend line.

Several features of TE-TTS already described in this report raise the possibility that TE-TTS can provide for cell type-specific regulation of gene expression. For example, the individual cases seen in Figure 1 clearly demonstrate cell type-specific termination of transcription by TEs. TEs also provide relatively more alternative TTS than non TE-TTS. Finally, individual TE-TTS are utilized less frequently than canonical known TTS from annotated gene models. In order to further investigate the potential genome-wide role of TE-TTS in the cell type-specific termination of transcription, we calculated cell type specificity levels of all TTS found in genes that are actively transcribed in at least three cell types. The cell type specificity metric we use measures the extent to which TTS are utilized at different levels across different cell types (see Methods). Internal TE-TTS show far greater levels of cell type specificity in the termination of transcription than seen for canonical TTS (Figure 7a). In addition, TE-TTS differ in their cell type-specific utilization based on their locations within human genes. Internal TE-TTS, which yield transcripts with truncated ORFs, show significantly more cell type-specific utilization than TE-TTS located in 3^′^ UTRs or downstream of canonical TTS, with internal non-TE-TTS showing similar specificity. The relatively highly cell type-specific utilization of internal TE-TTS suggests that they provide a mechanism for dynamic post-transcriptional regulation of human genes via the production of truncated transcripts. TE-TTS within 3^′^UTRs and downstream of canonical TTS are also generally more cell type-specific than canonical TTS, though to a lesser extent, and these TTS may be functional in producing longer or shorter 3^′^UTRs. As discussed previously, variation in 3^′^ UTR length provides for yet another level of post-transcriptional regulation [2,27].

The apparent cell type specificity of many TE-TTS suggests the possibility that the TE-TTS discovered in this study via the analysis of eight ENCODE cell types represent only a fraction of the total complement of TE-TTS that exist in the human genome. To address this possibility, we computed a rarefaction curve for TE-TTS by calculating the number of unique TE-TTS found using all possible combinations of 1–8 of the cell types analyzed here (Figure 7b). We then fit this rarefaction curve with a logarithmic trend line (y = 31.34lnx + 33.61; *r* = 0.99) to evaluate the extent to which the percent of detected TE-TTS is expected to change with increasing numbers of cell types. Based on the observed trend, we estimated that doubling the number of cell types included in a study of this kind would result in only a 20% increase in the number of TE-TTS found, suggesting a substantially diminishing rate of returns with respect to the discovery of novel TE-TTS as more cell types are added. Similarly, the number of genes found to contain a TE-TTS leveled off as more cell types were included. Nevertheless, taking 210 as the total number of human cell types indicates that the TE-TTS discovered here represent half of the total number of human gene TE-TTS. Thus, TEs may provide close to 20,000 TE-TTS for ~11,000 human genes.

## Conclusions

### Transcription termination as the origin of TE antisense orientation bias

It has been appreciated for some time that TE sequences within the introns of human genes show a strong antisense orientation bias [19,28]. It was proposed that this bias is due to the propensity of the TE sequences to terminate transcription of host genes when inserted in the sense orientation, resulting in selection against such sense-oriented insertions [29]. Nevertheless, studies to date on the ability of TEs to terminate transcription have not revealed evidence in support of this hypothesis [11,12]. Here, for the first time, we provide genome-scale evidence in support of the notion that the antisense orientation bias of TEs can be attributed to their ability to preferentially terminate host gene transcription when inserted in the sense orientation. We have shown that TE sequences that provide a TTS are significantly more likely to be found in the sense orientation than other intragenic TE sequences (Figure 3) and that TE-TTS in the sense orientation terminate transcription much more efficiently than those found in the antisense orientation (Figure 6). Nevertheless, there may be additional as yet unknown factors that also contribute to the observed antisense orientation of human TEs.

Among the eight TE families studied here, the Alu, ERV and MaLR families are distinct from the other five families. They all exert substantial effects on the expression of human genes via the termination of transcription, but they do so using distinct genome-wide metastrategies. TTS derived from Alu sequences are generally weakly utilized compared to other TE families, while at the same time having a weak antisense orientation bias. The weaker orientation bias of Alu sequences suggests that there is weaker selection against Alu sequences inserted in sense. We suggest that this weaker selection is due to the generally weak utilization of Alu-TTS. Conversely, LTR elements, the ERV and MaLR families, show a very strong antisense bias and a strong utilization; such strong utilization may account for the strong antisense orientation of LTR elements. The Alu family, by providing many relatively weak TTS, can affect a large number of genes, albeit in a subtle way on a gene-by-gene basis, whereas LTR elements have much larger effects on the expression levels of a smaller number of genes.

### Cell type- and lineage-specific termination of transcription by TEs

Evidence reported here points to the contribution of TE sequences to the cell type-specific termination of transcription; we have shown that internal TTS derived from TE sequences are significantly more cell type-specific compared to canonical TTS (Figure 7a). In this way, TE sequences have contributed substantially to the generation of cell type-specific patterns of human gene expression via the pre-mature termination of transcription. In addition to providing for cell type-specific termination of transcription, data reported here indicate that TE sequences are also likely to have contributed substantially to evolutionary lineage-specific transcription termination. Numerous TE insertions can be generated in a short evolutionary time, and accordingly the majority of human TE subfamilies are lineage-specific [21]. This means that the regulatory effects that these TEs exert on their host genomes, including termination of transcription as shown here, will also be lineage-specific and account for regulatory differences between evolutionary lineages.

The Alu family, for example, is a relatively young family of TEs, which is confined to the primate evolutionary lineage. The Alu family has been active throughout primate evolution [4] and has likely been altering primate gene expression via TTS donation since the origin of the primate lineage, as can be seen from the results on the more ancient Alu antecedents from the FAM-related subfamilies (Figure 5). This process appears to have accelerated, leading to even more species-specific differences in transcription termination, with the amplification of the more modern Alu dimers (Figure 5).

### Transcription termination via TE sequences as a common phenomenon

The abundance of TE insertions across eukaryotic lineages suggests that the effect of TE insertions on gene expression via the termination of transcription is not limited to humans [30]. In this study, we characterized the involvement of eight evolutionary diverse families of TEs in the termination of transcription. TEs related to these eight families are present in the genomes of many other eukaryotes. For instance, while LTR elements are functionally dead in humans [4], multiple LTR element families are still highly active in other species, *e.g.,* the intracisternal A particle (IAP) family of mouse. Indeed, it has been estimated that 10% of mutations in mouse are caused by the novel retrotransposition of an LTR element. As a consequence of this, mice presumably have to contend with a great deal of deleterious transcription termination via novel LTR element insertions. However, these novel insertions also provide the opportunity for innovation in the regulation of gene expression.

## Methods

### Characterization of transcription termination sites (TTS)

Mappings of ENCODE PET data from the GM12878, H1HESC, HepG2, HeLaS3, HUVEC, K562, NHEK and prostate cell types were downloaded from the ENCODE repository on the UCSC genome browser for the hg18 version of the human genome [14,15]. PET data from nucleus (GM12878, HepG2, HeLaS3, HUVEC, K562 and NHEK) or whole-cell (H1HESC and prostate) were used to characterize TTS. PET 3′-ends from the same data set that overlapped or were separated by 20 or fewer bases were taken as putative TTS clusters. Only those TTS clusters that had a normalized PET tag count of at least 20 per 10 million, tags mapped in at least one cell type were considered for further analysis. For these clusters, the specific locations of the TTS for each cluster were taken to be the base with the highest density of mapped PET 3^′^-ends. TTS clusters across different cell types that overlapped by at least 80% were taken to be the same TTS.

UCSC gene model annotations [31] were used to associate TTS defined in this way with known human genes. A TTS was considered to be associated with a gene if the linked 5^′^ ends of the PET tags were mapped to the annotated promoter of the gene and the linked 3^′^ end TTS cluster was found within the annotated transcriptional united or up to 5-kb downstream of the canonical annotated TTS. Human gene TTS characterized in this way were then co-located with TE sequences using the RepeatMasker annotations [23]. As it has been previously shown that transcription termination occurs within 50 bp of the polyadenylation signal [32], TE-TTS were defined as those TTS clusters for which the peak base was at least 50 bp downstream from the start of a TE insertion and less than 15 bp downstream of the end of the insertion.

### Histone modification enrichment analysis

The chromatin environment of PET-characterized TTS was characterized using ENCODE ChIP-seq data [33]. Where available for the same cell types as the PET data, ChIP-seq reads for the H3K9Ac, H3K27Me3 and H3K36Me3 modifications that were downloaded from the ENCODE repository on the UCSC genome browser [15,16] were mapped to the human genome reference sequence (UCSC hg18; NCBI build 36.1) using the Bowtie short read alignment utility [34]. Tags that mapped to multiple locations were resolved using the GibbsAM utility [35]. The average numbers of ChIP-seq tags were found in five base-pair windows ±5 kb of (1) TE-derived TTS, (2) intragenic TE insertions that do not provide a TTS and (3) non-TE-derived TTS.

### Utilization of PET-characterized TTS

TTS for which the region including the TTS had a normalized PET tag count of at least 20 were designated transcribed. From each cell type, those regions that were in the top 75% most transcribed, as calculated using PET tag counts, in that cell type were designated as actively transcribed. For both TE-TTS and non-TE-TTS, the utilization of actively transcribed TTS in a cell type was determined by first determining the number of PET tags that begin upstream of the TTS and that terminate in the TTS or downstream of the TTS. The utilization was then calculated using the following formula:

$$utilization=\frac{reads\;terminated}{reads\;terminated+reads\;passing}$$

### Cell type specificity of TE-derived TTS

In order to consider only relatively strong and highly utilized TE-TTS, a TTS was considered for differential utilization if the TTS (1) had a strength of utilization of at least 20% in at least one cell type and (2) the region was actively transcribed (as described above) in at least three cell types. The cell type specificity of a given TE-TTS was calculated using the following formula:

$$cell\;type-specificity=\frac{\sum_{i=1}^{cell\;types-1}\;MAX\left(utilization\right)-utilization_{i}}{cell\;types-1}/MAX\left(utilization\right)$$

### Estimation of the total number of TE-TTS and genes with TE-TTS

To estimate the upper bound for the number of TE-derived TTS in the human genome, we found, for all possible combinations of the eight cell types used here, the number of TE-derived TTS found with each combination. A logarithmic trend line was used to estimate the number of TE-derived TTS that would be found with increasing numbers of cell types. The same analysis was applied for the total number of human genes that bear at least one TE-TTS.

## Abbreviations

TE: Transposable element; TTS: Transcription termination site; TE-TTS: Transposable element-derived transcription termination site; PET: Paired-end ditag; LTR: Long terminal repeat; MIR: Mammalian interspersed repeat; MaLR: Mammamlian apparent LTR; ChIP-seq: Chromatin immunoprecipitation followed by high-throughput sequencing.

## Competing interests

The authors declare that they have no competing interest.

## Authors’ contributions

ABC and IKJ conceived of the study. ABC designed and carried out the analysis. ABC and IKJ wrote the manuscript. Both authors read and approved the final manuscript.

## Supplementary Material

Additional file 1 Supplementary material. Click here for file
