# Supplementary material for: Cell type-specific termination of transcription by transposable element sequences
Source: Mob DNA. 2012 Sep 30;3:15. doi: 10.1186/1759-8753-3-15 (PMC3517506; doi:10.1186/1759-8753-3-15)
Supplement: Additional file 1 — Supplementary material. [file 1759-8753-3-15-S1.pptx]

## Slide 1
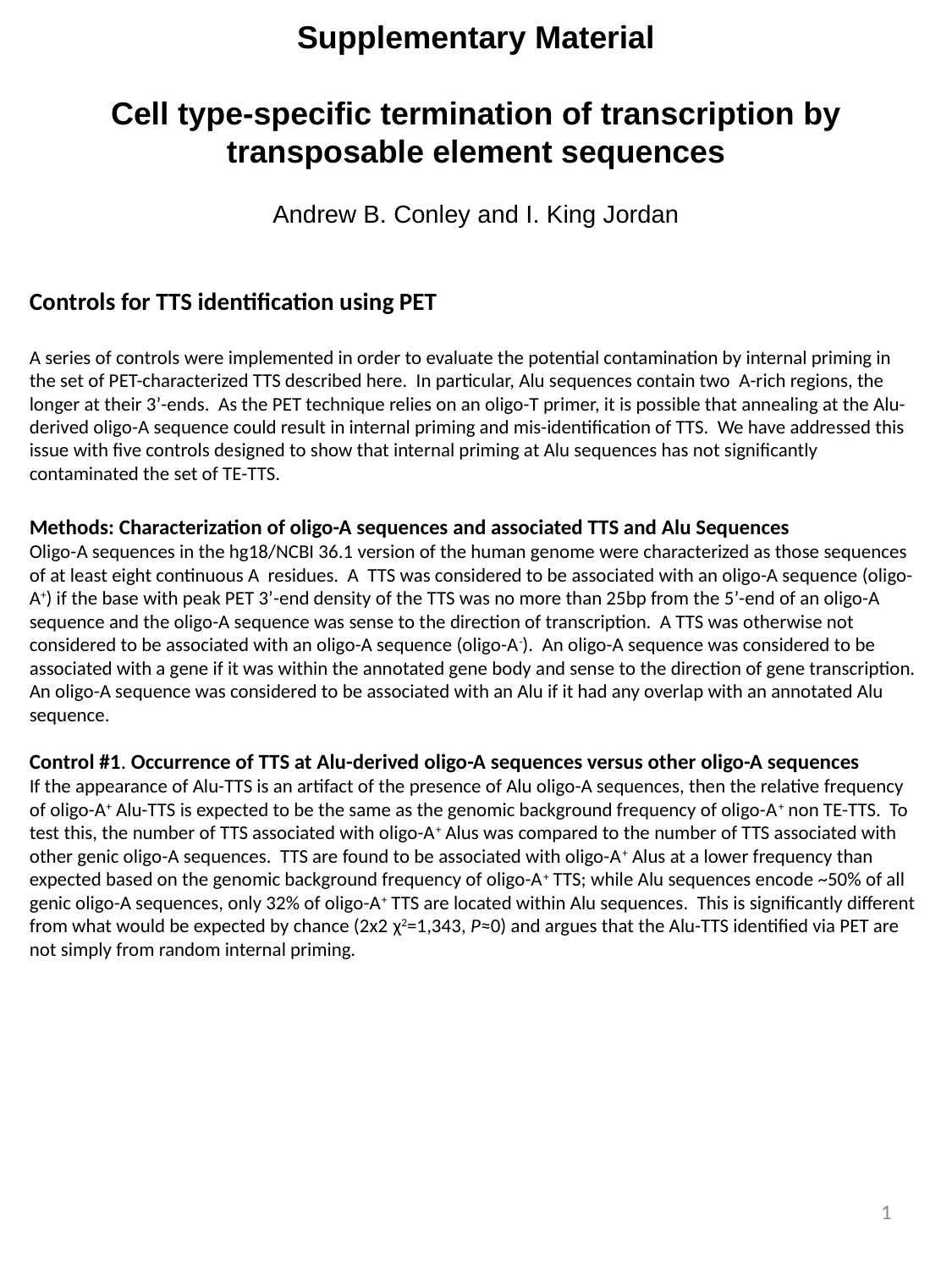

Supplementary Material
Cell type-specific termination of transcription by transposable element sequences
Andrew B. Conley and I. King Jordan
Controls for TTS identification using PET
A series of controls were implemented in order to evaluate the potential contamination by internal priming in the set of PET-characterized TTS described here. In particular, Alu sequences contain two A-rich regions, the longer at their 3’-ends. As the PET technique relies on an oligo-T primer, it is possible that annealing at the Alu-derived oligo-A sequence could result in internal priming and mis-identification of TTS. We have addressed this issue with five controls designed to show that internal priming at Alu sequences has not significantly contaminated the set of TE-TTS.
Methods: Characterization of oligo-A sequences and associated TTS and Alu Sequences
Oligo-A sequences in the hg18/NCBI 36.1 version of the human genome were characterized as those sequences of at least eight continuous A residues. A TTS was considered to be associated with an oligo-A sequence (oligo-A+) if the base with peak PET 3’-end density of the TTS was no more than 25bp from the 5’-end of an oligo-A sequence and the oligo-A sequence was sense to the direction of transcription. A TTS was otherwise not considered to be associated with an oligo-A sequence (oligo-A-). An oligo-A sequence was considered to be associated with a gene if it was within the annotated gene body and sense to the direction of gene transcription. An oligo-A sequence was considered to be associated with an Alu if it had any overlap with an annotated Alu sequence.
Control #1. Occurrence of TTS at Alu-derived oligo-A sequences versus other oligo-A sequences
If the appearance of Alu-TTS is an artifact of the presence of Alu oligo-A sequences, then the relative frequency of oligo-A+ Alu-TTS is expected to be the same as the genomic background frequency of oligo-A+ non TE-TTS. To test this, the number of TTS associated with oligo-A+ Alus was compared to the number of TTS associated with other genic oligo-A sequences. TTS are found to be associated with oligo-A+ Alus at a lower frequency than expected based on the genomic background frequency of oligo-A+ TTS; while Alu sequences encode ~50% of all genic oligo-A sequences, only 32% of oligo-A+ TTS are located within Alu sequences. This is significantly different from what would be expected by chance (2x2 χ2=1,343, P≈0) and argues that the Alu-TTS identified via PET are not simply from random internal priming.
1

## Slide 2
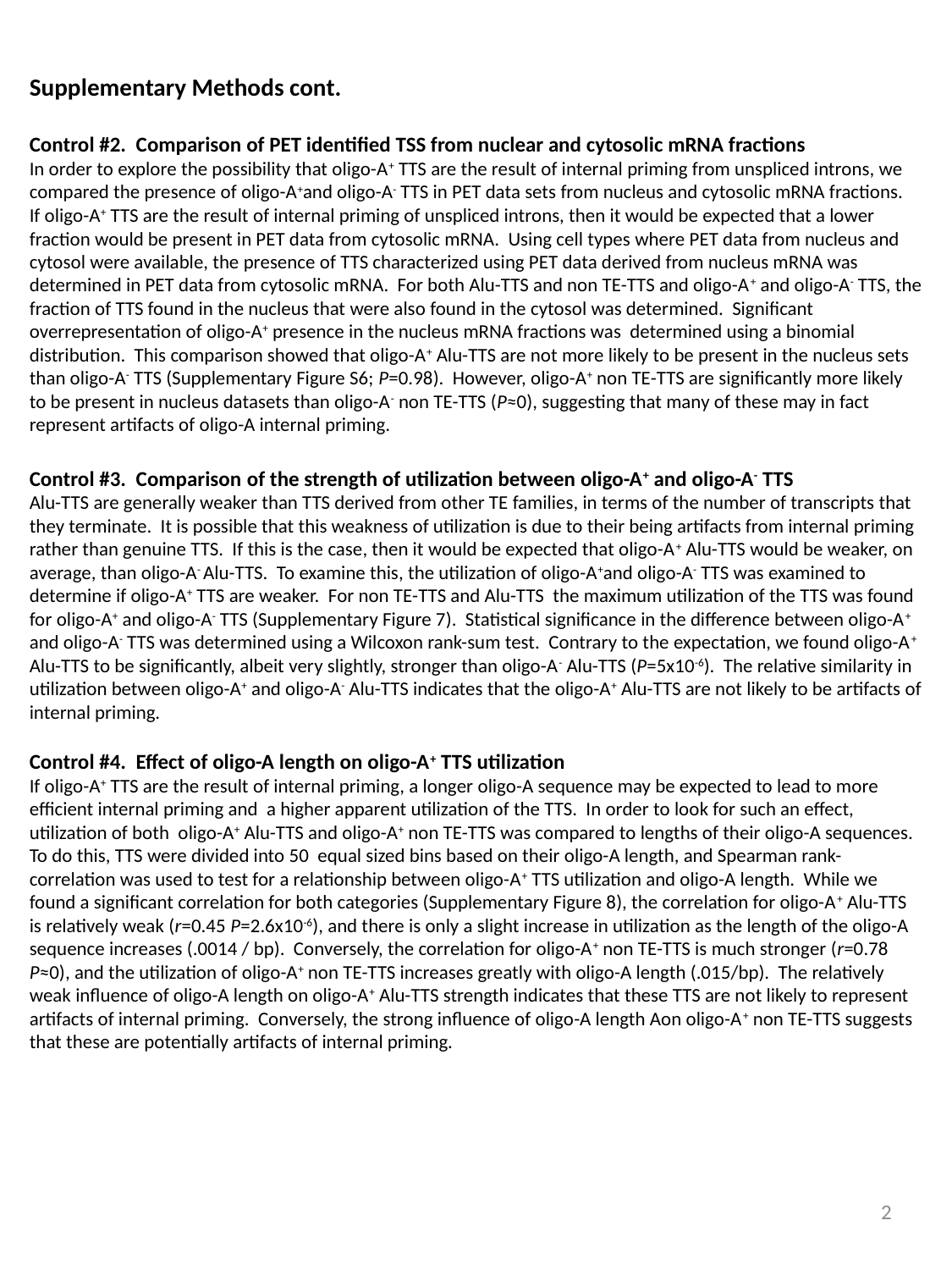

Supplementary Methods cont.
Control #2. Comparison of PET identified TSS from nuclear and cytosolic mRNA fractions
In order to explore the possibility that oligo-A+ TTS are the result of internal priming from unspliced introns, we compared the presence of oligo-A+and oligo-A- TTS in PET data sets from nucleus and cytosolic mRNA fractions. If oligo-A+ TTS are the result of internal priming of unspliced introns, then it would be expected that a lower fraction would be present in PET data from cytosolic mRNA. Using cell types where PET data from nucleus and cytosol were available, the presence of TTS characterized using PET data derived from nucleus mRNA was determined in PET data from cytosolic mRNA. For both Alu-TTS and non TE-TTS and oligo-A+ and oligo-A- TTS, the fraction of TTS found in the nucleus that were also found in the cytosol was determined. Significant overrepresentation of oligo-A+ presence in the nucleus mRNA fractions was determined using a binomial distribution. This comparison showed that oligo-A+ Alu-TTS are not more likely to be present in the nucleus sets than oligo-A- TTS (Supplementary Figure S6; P=0.98). However, oligo-A+ non TE-TTS are significantly more likely to be present in nucleus datasets than oligo-A- non TE-TTS (P≈0), suggesting that many of these may in fact represent artifacts of oligo-A internal priming.
Control #3. Comparison of the strength of utilization between oligo-A+ and oligo-A- TTS
Alu-TTS are generally weaker than TTS derived from other TE families, in terms of the number of transcripts that they terminate. It is possible that this weakness of utilization is due to their being artifacts from internal priming rather than genuine TTS. If this is the case, then it would be expected that oligo-A+ Alu-TTS would be weaker, on average, than oligo-A- Alu-TTS. To examine this, the utilization of oligo-A+and oligo-A- TTS was examined to determine if oligo-A+ TTS are weaker. For non TE-TTS and Alu-TTS the maximum utilization of the TTS was found for oligo-A+ and oligo-A- TTS (Supplementary Figure 7). Statistical significance in the difference between oligo-A+ and oligo-A- TTS was determined using a Wilcoxon rank-sum test. Contrary to the expectation, we found oligo-A+ Alu-TTS to be significantly, albeit very slightly, stronger than oligo-A- Alu-TTS (P=5x10^-6^). The relative similarity in utilization between oligo-A+ and oligo-A- Alu-TTS indicates that the oligo-A+ Alu-TTS are not likely to be artifacts of internal priming.
Control #4. Effect of oligo-A length on oligo-A+ TTS utilization
If oligo-A+ TTS are the result of internal priming, a longer oligo-A sequence may be expected to lead to more efficient internal priming and a higher apparent utilization of the TTS. In order to look for such an effect, utilization of both oligo-A+ Alu-TTS and oligo-A+ non TE-TTS was compared to lengths of their oligo-A sequences. To do this, TTS were divided into 50 equal sized bins based on their oligo-A length, and Spearman rank-correlation was used to test for a relationship between oligo-A+ TTS utilization and oligo-A length. While we found a significant correlation for both categories (Supplementary Figure 8), the correlation for oligo-A+ Alu-TTS is relatively weak (r=0.45 P=2.6x10^-6^), and there is only a slight increase in utilization as the length of the oligo-A sequence increases (.0014 / bp). Conversely, the correlation for oligo-A+ non TE-TTS is much stronger (r=0.78 P≈0), and the utilization of oligo-A+ non TE-TTS increases greatly with oligo-A length (.015/bp). The relatively weak influence of oligo-A length on oligo-A+ Alu-TTS strength indicates that these TTS are not likely to represent artifacts of internal priming. Conversely, the strong influence of oligo-A length Aon oligo-A+ non TE-TTS suggests that these are potentially artifacts of internal priming.
2

## Slide 3
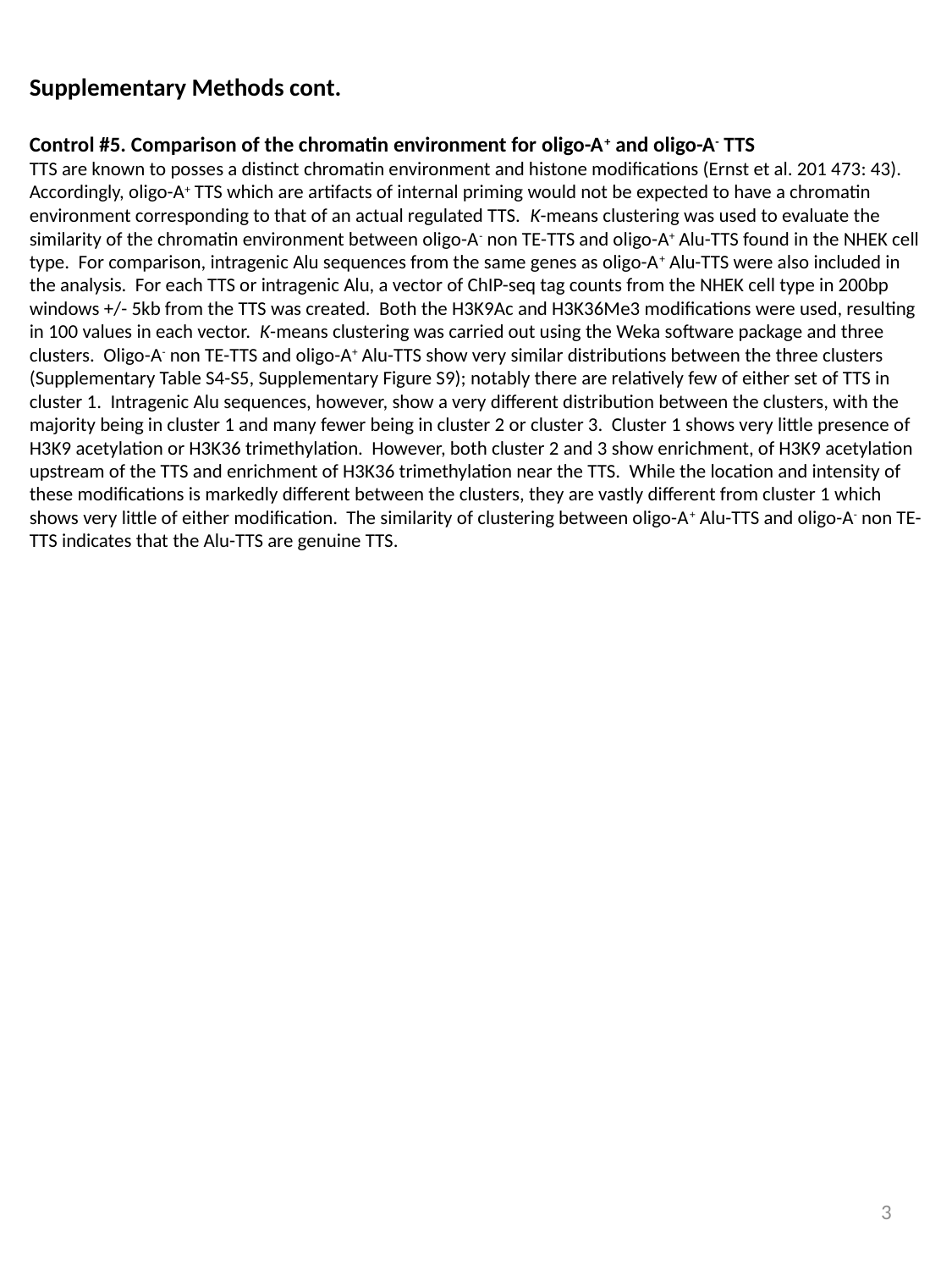

Supplementary Methods cont.
Control #5. Comparison of the chromatin environment for oligo-A+ and oligo-A- TTS
TTS are known to posses a distinct chromatin environment and histone modifications (Ernst et al. 201 473: 43). Accordingly, oligo-A+ TTS which are artifacts of internal priming would not be expected to have a chromatin environment corresponding to that of an actual regulated TTS. K-means clustering was used to evaluate the similarity of the chromatin environment between oligo-A- non TE-TTS and oligo-A+ Alu-TTS found in the NHEK cell type. For comparison, intragenic Alu sequences from the same genes as oligo-A+ Alu-TTS were also included in the analysis. For each TTS or intragenic Alu, a vector of ChIP-seq tag counts from the NHEK cell type in 200bp windows +/- 5kb from the TTS was created. Both the H3K9Ac and H3K36Me3 modifications were used, resulting in 100 values in each vector. K-means clustering was carried out using the Weka software package and three clusters. Oligo-A- non TE-TTS and oligo-A+ Alu-TTS show very similar distributions between the three clusters (Supplementary Table S4-S5, Supplementary Figure S9); notably there are relatively few of either set of TTS in cluster 1. Intragenic Alu sequences, however, show a very different distribution between the clusters, with the majority being in cluster 1 and many fewer being in cluster 2 or cluster 3. Cluster 1 shows very little presence of H3K9 acetylation or H3K36 trimethylation. However, both cluster 2 and 3 show enrichment, of H3K9 acetylation upstream of the TTS and enrichment of H3K36 trimethylation near the TTS. While the location and intensity of these modifications is markedly different between the clusters, they are vastly different from cluster 1 which shows very little of either modification. The similarity of clustering between oligo-A+ Alu-TTS and oligo-A- non TE-TTS indicates that the Alu-TTS are genuine TTS.
3

## Slide 4
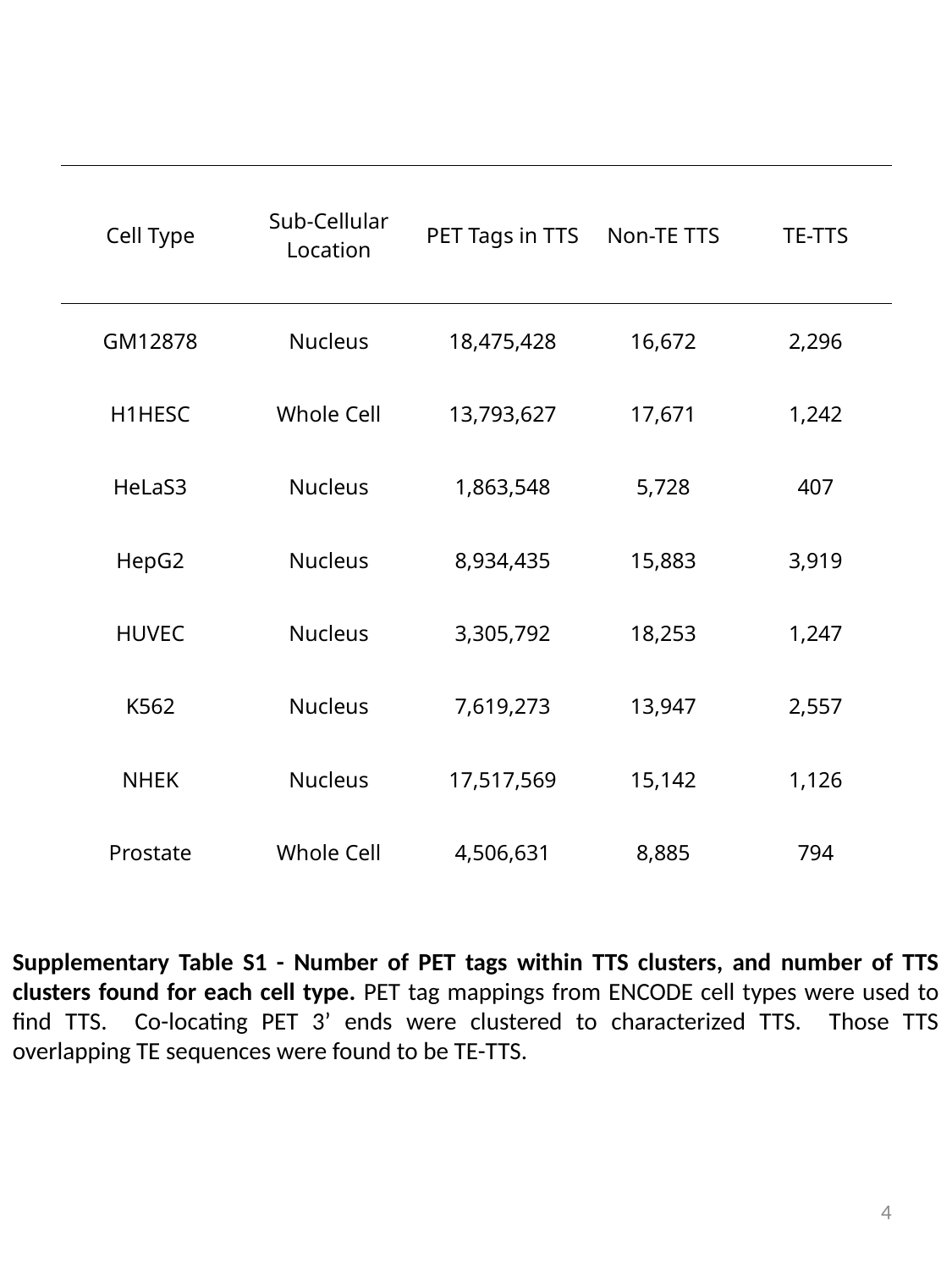

| Cell Type | Sub-Cellular Location | PET Tags in TTS | Non-TE TTS | TE-TTS |
| --- | --- | --- | --- | --- |
| GM12878 | Nucleus | 18,475,428 | 16,672 | 2,296 |
| H1HESC | Whole Cell | 13,793,627 | 17,671 | 1,242 |
| HeLaS3 | Nucleus | 1,863,548 | 5,728 | 407 |
| HepG2 | Nucleus | 8,934,435 | 15,883 | 3,919 |
| HUVEC | Nucleus | 3,305,792 | 18,253 | 1,247 |
| K562 | Nucleus | 7,619,273 | 13,947 | 2,557 |
| NHEK | Nucleus | 17,517,569 | 15,142 | 1,126 |
| Prostate | Whole Cell | 4,506,631 | 8,885 | 794 |
Supplementary Table S1 - Number of PET tags within TTS clusters, and number of TTS clusters found for each cell type. PET tag mappings from ENCODE cell types were used to find TTS. Co-locating PET 3’ ends were clustered to characterized TTS. Those TTS overlapping TE sequences were found to be TE-TTS.
4

## Slide 5
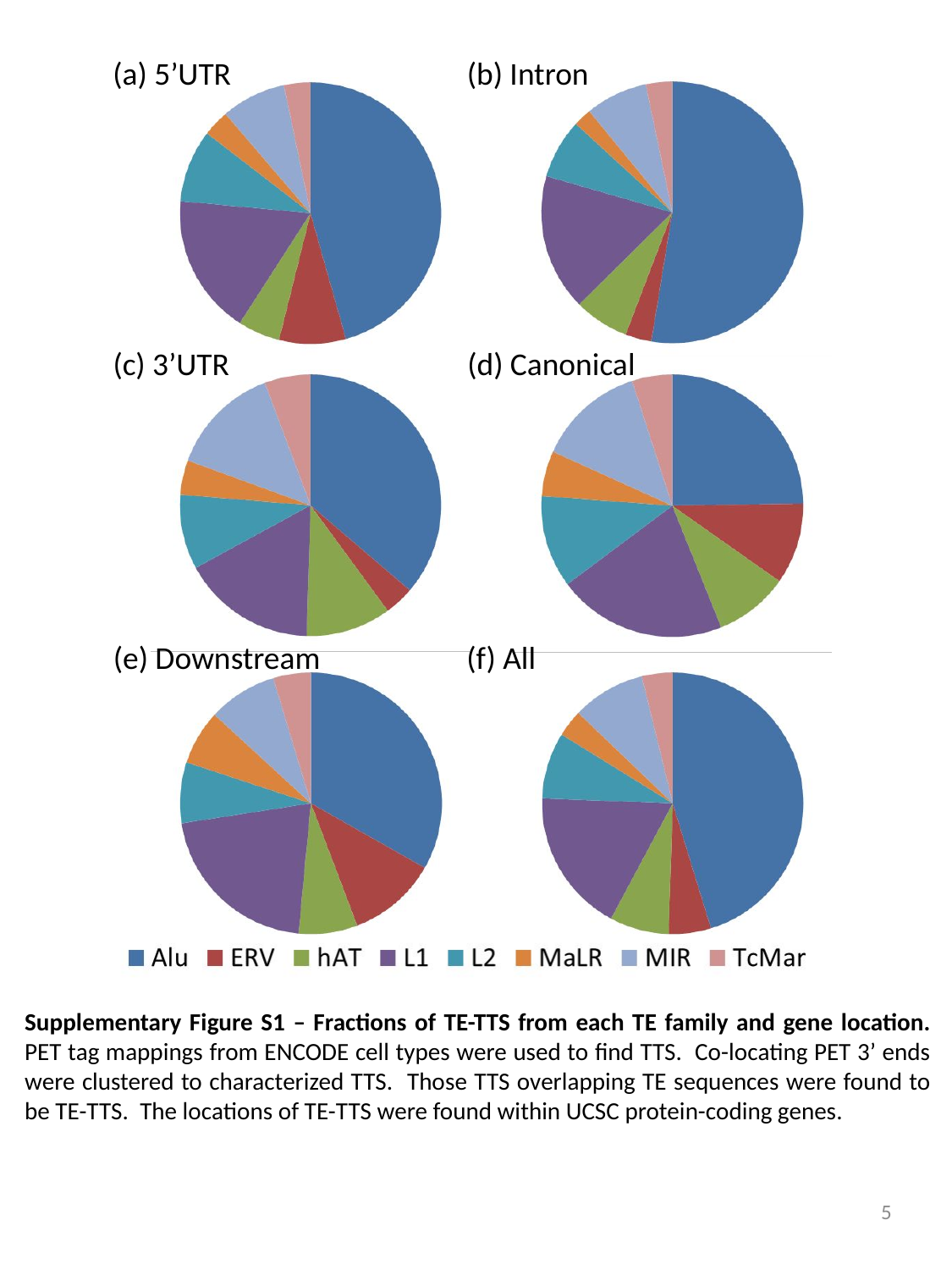

(a) 5’UTR
(b) Intron
(c) 3’UTR
(d) Canonical
(e) Downstream
(f) All
Supplementary Figure S1 – Fractions of TE-TTS from each TE family and gene location. PET tag mappings from ENCODE cell types were used to find TTS. Co-locating PET 3’ ends were clustered to characterized TTS. Those TTS overlapping TE sequences were found to be TE-TTS. The locations of TE-TTS were found within UCSC protein-coding genes.
5

## Slide 6
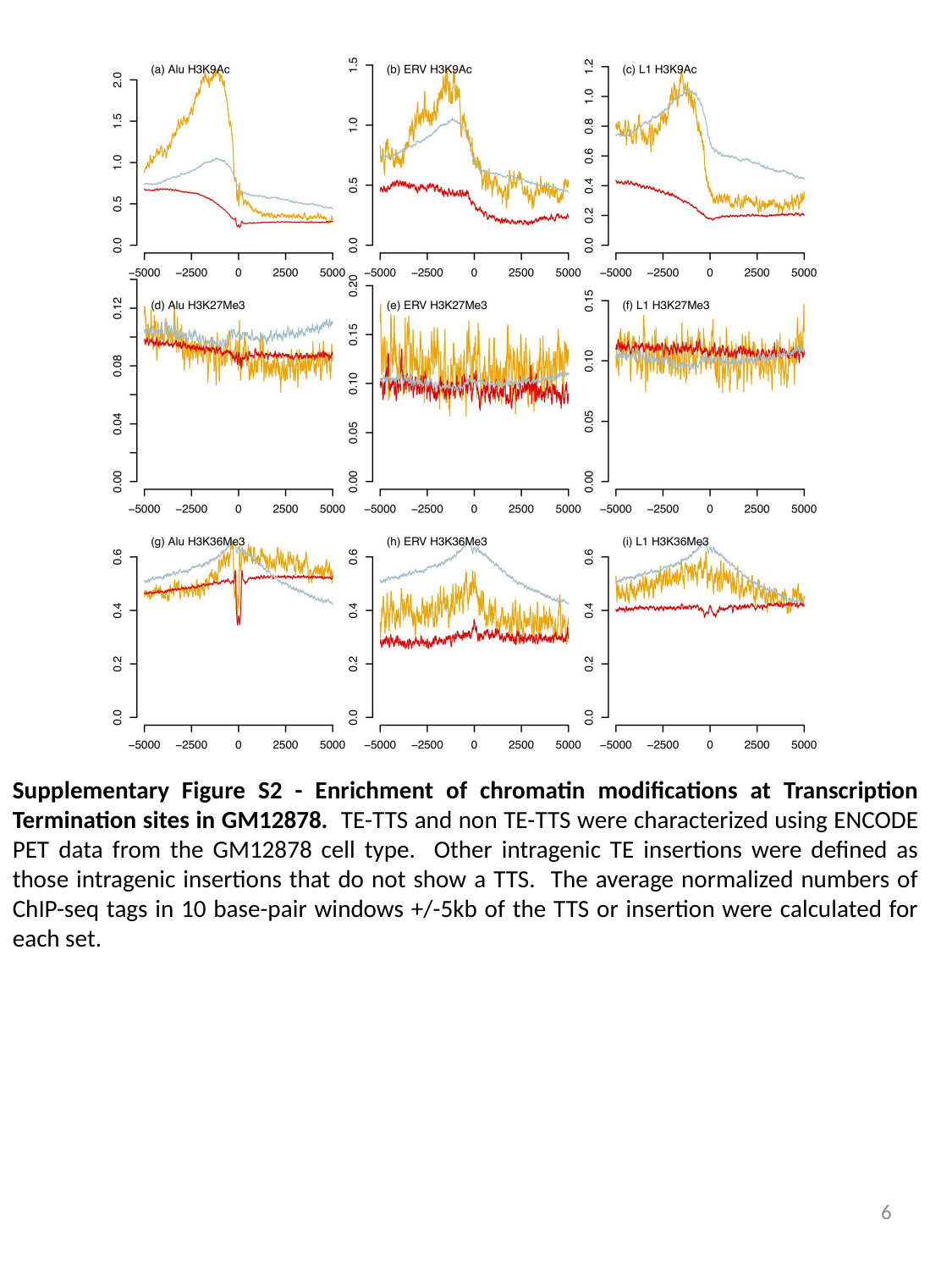

Supplementary Figure S2 - Enrichment of chromatin modifications at Transcription Termination sites in GM12878. TE-TTS and non TE-TTS were characterized using ENCODE PET data from the GM12878 cell type. Other intragenic TE insertions were defined as those intragenic insertions that do not show a TTS. The average normalized numbers of ChIP-seq tags in 10 base-pair windows +/-5kb of the TTS or insertion were calculated for each set.
6

## Slide 7
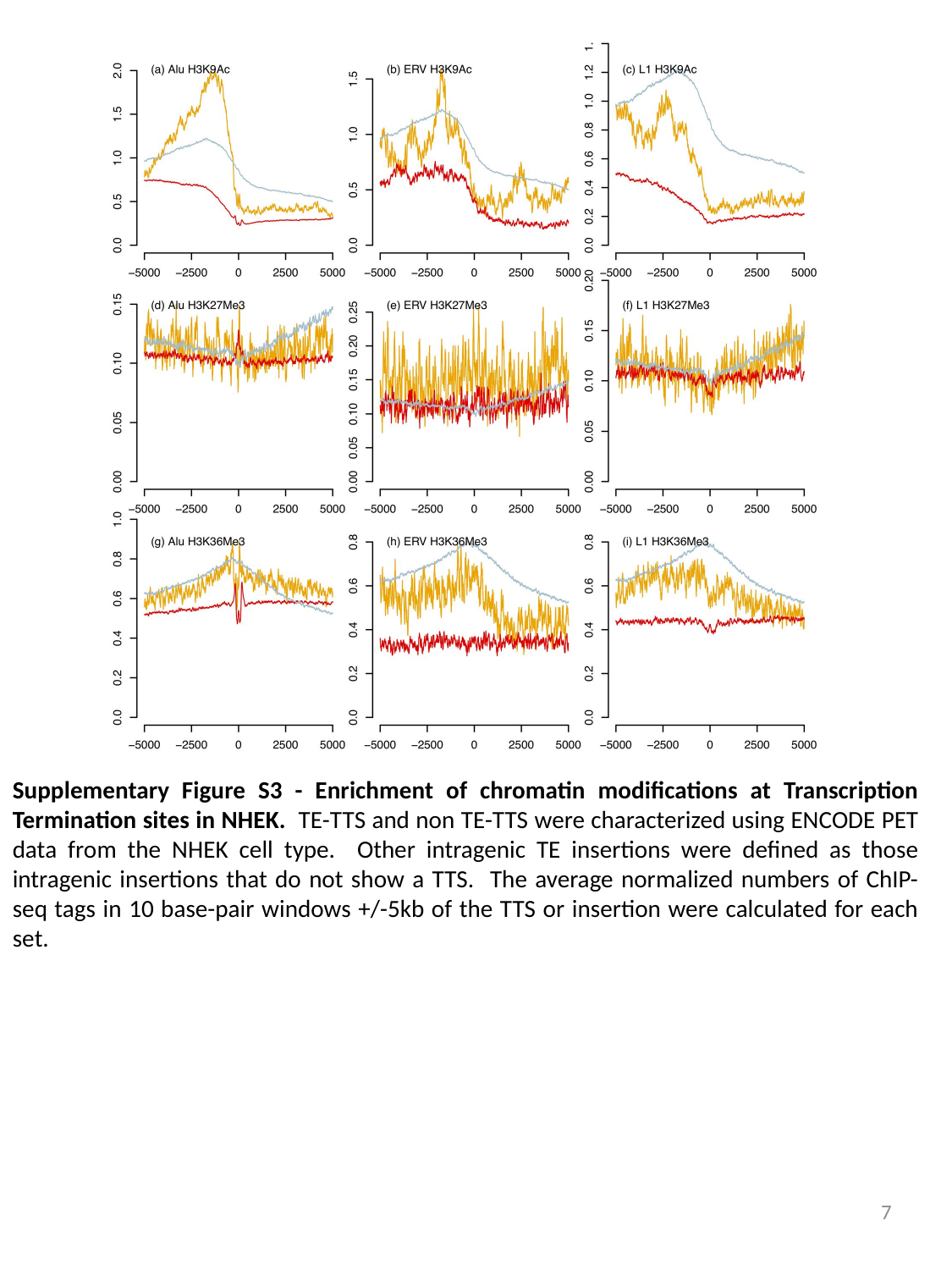

Supplementary Figure S3 - Enrichment of chromatin modifications at Transcription Termination sites in NHEK. TE-TTS and non TE-TTS were characterized using ENCODE PET data from the NHEK cell type. Other intragenic TE insertions were defined as those intragenic insertions that do not show a TTS. The average normalized numbers of ChIP-seq tags in 10 base-pair windows +/-5kb of the TTS or insertion were calculated for each set.
7

## Slide 8
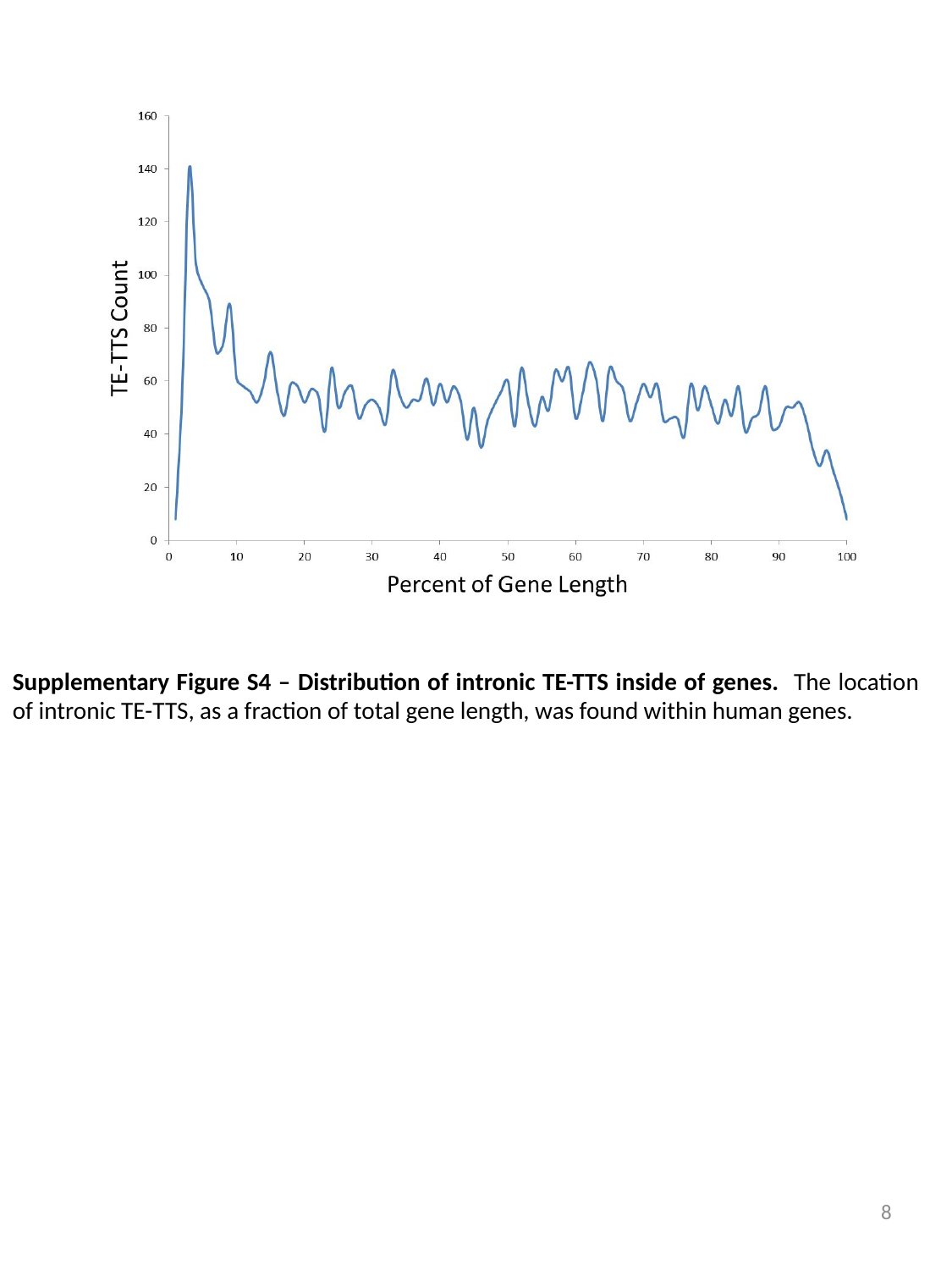

Supplementary Figure S4 – Distribution of intronic TE-TTS inside of genes. The location of intronic TE-TTS, as a fraction of total gene length, was found within human genes.
8

## Slide 9
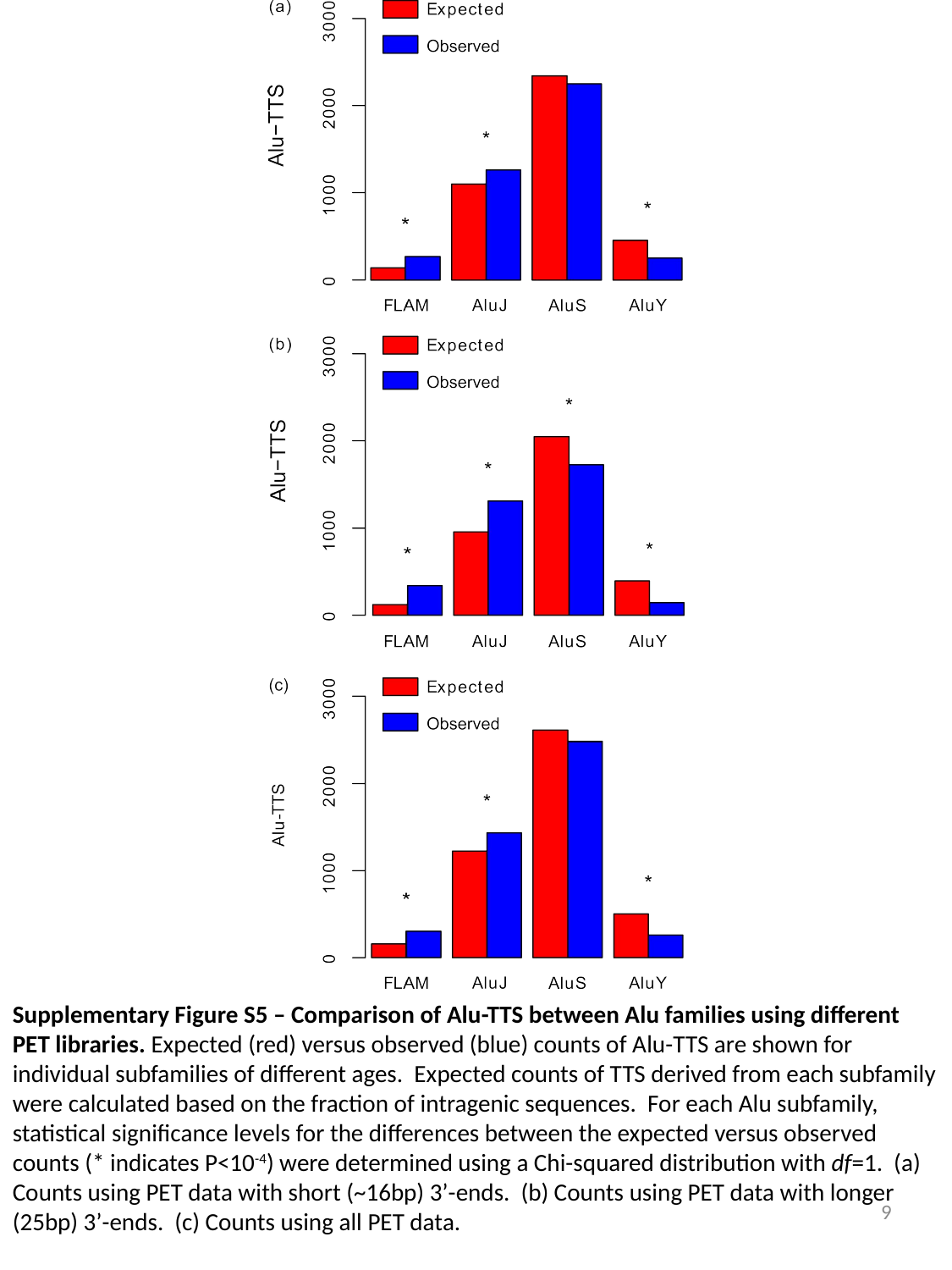

Supplementary Figure S5 – Comparison of Alu-TTS between Alu families using different PET libraries. Expected (red) versus observed (blue) counts of Alu-TTS are shown for individual subfamilies of different ages. Expected counts of TTS derived from each subfamily were calculated based on the fraction of intragenic sequences. For each Alu subfamily, statistical significance levels for the differences between the expected versus observed counts (* indicates P<10-4) were determined using a Chi-squared distribution with df=1. (a) Counts using PET data with short (~16bp) 3’-ends. (b) Counts using PET data with longer (25bp) 3’-ends. (c) Counts using all PET data.
9

## Slide 10
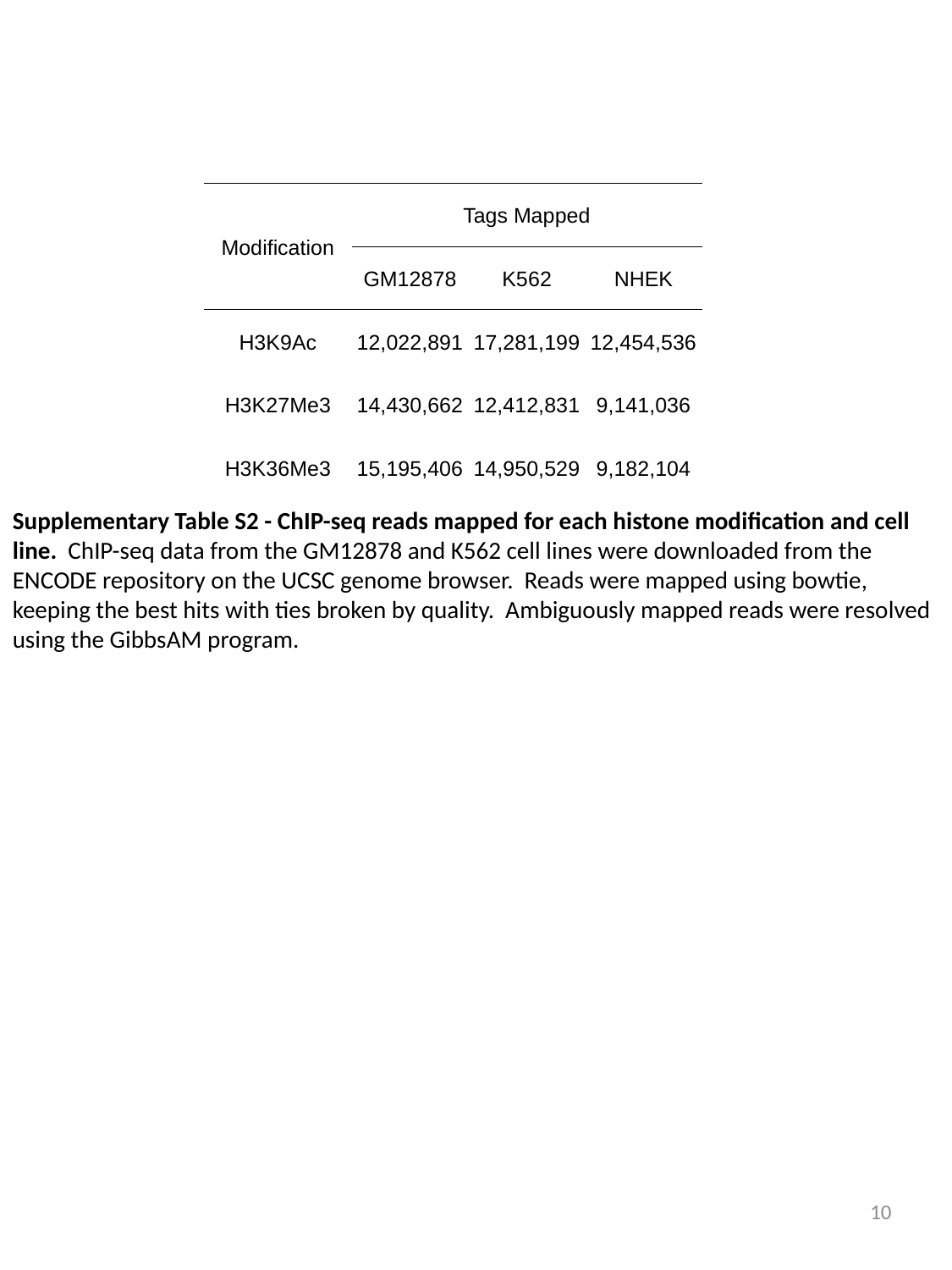

| Modification | Tags Mapped | | |
| --- | --- | --- | --- |
| | GM12878 | K562 | NHEK |
| H3K9Ac | 12,022,891 | 17,281,199 | 12,454,536 |
| H3K27Me3 | 14,430,662 | 12,412,831 | 9,141,036 |
| H3K36Me3 | 15,195,406 | 14,950,529 | 9,182,104 |
Supplementary Table S2 - ChIP-seq reads mapped for each histone modification and cell line. ChIP-seq data from the GM12878 and K562 cell lines were downloaded from the ENCODE repository on the UCSC genome browser. Reads were mapped using bowtie, keeping the best hits with ties broken by quality. Ambiguously mapped reads were resolved using the GibbsAM program.
10

## Slide 11
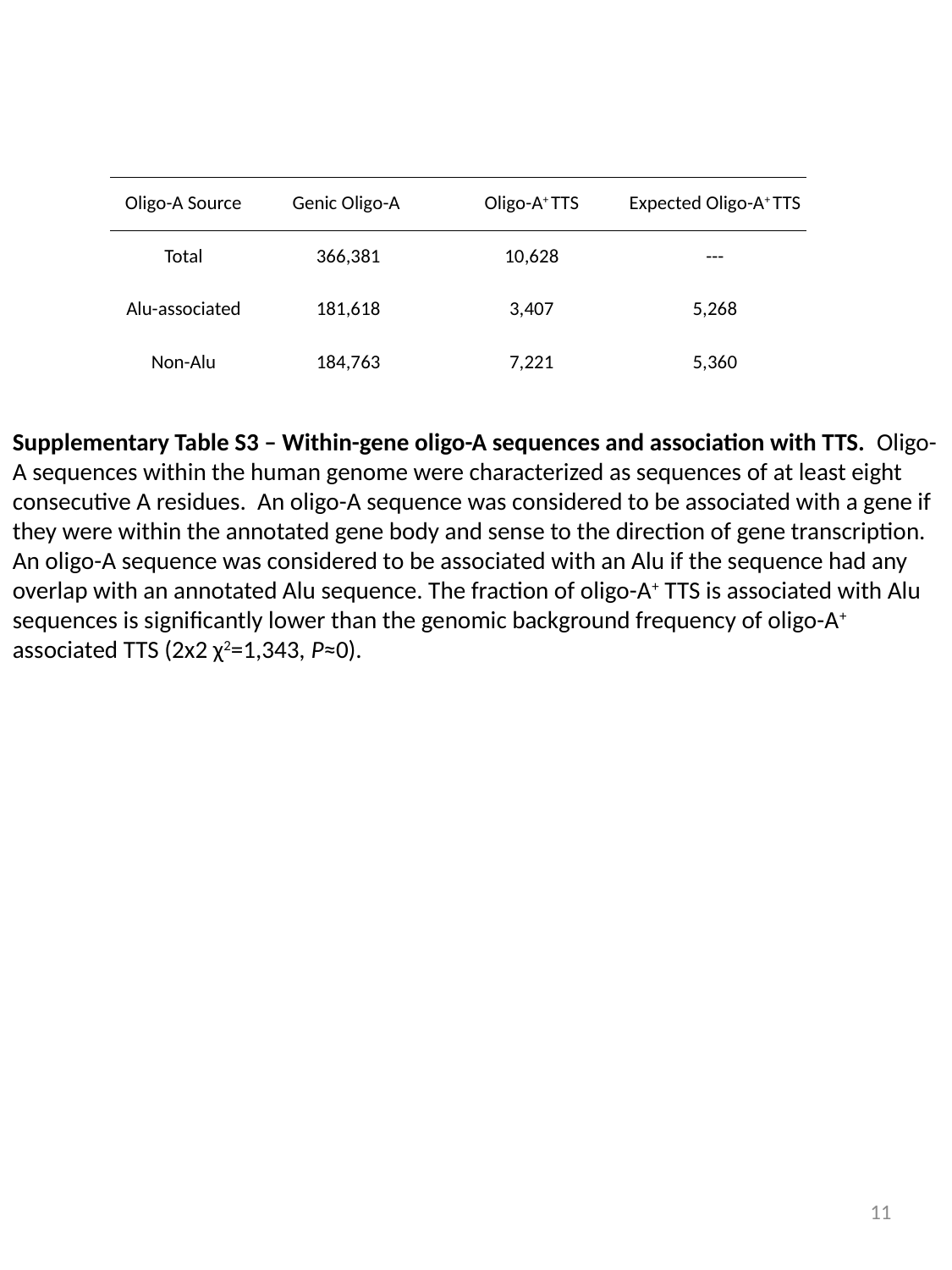

| Oligo-A Source | Genic Oligo-A | Oligo-A+ TTS | Expected Oligo-A+ TTS |
| --- | --- | --- | --- |
| Total | 366,381 | 10,628 | --- |
| Alu-associated | 181,618 | 3,407 | 5,268 |
| Non-Alu | 184,763 | 7,221 | 5,360 |
Supplementary Table S3 – Within-gene oligo-A sequences and association with TTS. Oligo-A sequences within the human genome were characterized as sequences of at least eight consecutive A residues. An oligo-A sequence was considered to be associated with a gene if they were within the annotated gene body and sense to the direction of gene transcription. An oligo-A sequence was considered to be associated with an Alu if the sequence had any overlap with an annotated Alu sequence. The fraction of oligo-A+ TTS is associated with Alu sequences is significantly lower than the genomic background frequency of oligo-A+ associated TTS (2x2 χ2=1,343, P≈0).
11

## Slide 12
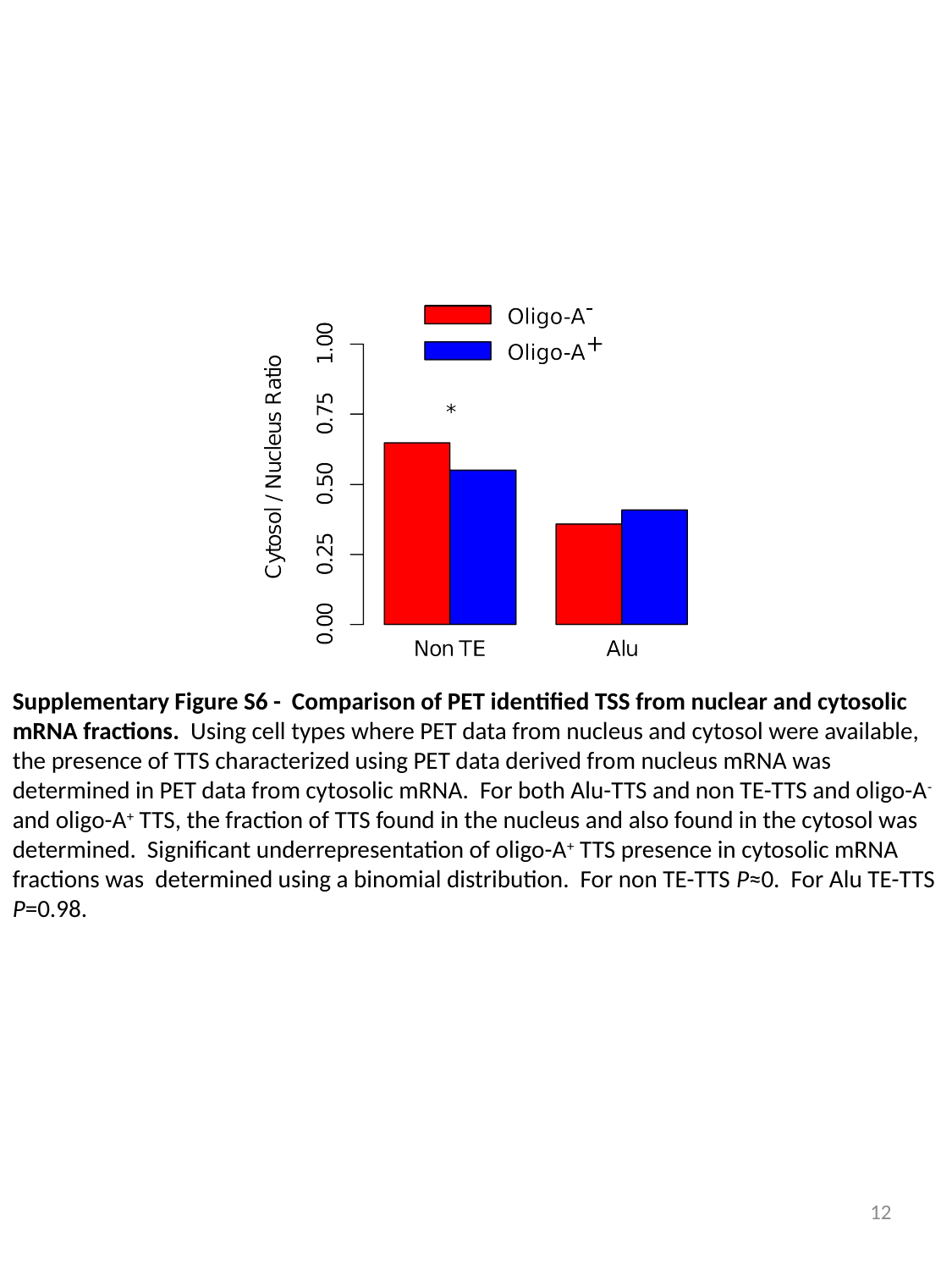

Supplementary Figure S6 - Comparison of PET identified TSS from nuclear and cytosolic mRNA fractions. Using cell types where PET data from nucleus and cytosol were available, the presence of TTS characterized using PET data derived from nucleus mRNA was determined in PET data from cytosolic mRNA. For both Alu-TTS and non TE-TTS and oligo-A- and oligo-A+ TTS, the fraction of TTS found in the nucleus and also found in the cytosol was determined. Significant underrepresentation of oligo-A+ TTS presence in cytosolic mRNA fractions was determined using a binomial distribution. For non TE-TTS P≈0. For Alu TE-TTS P=0.98.
12

## Slide 13
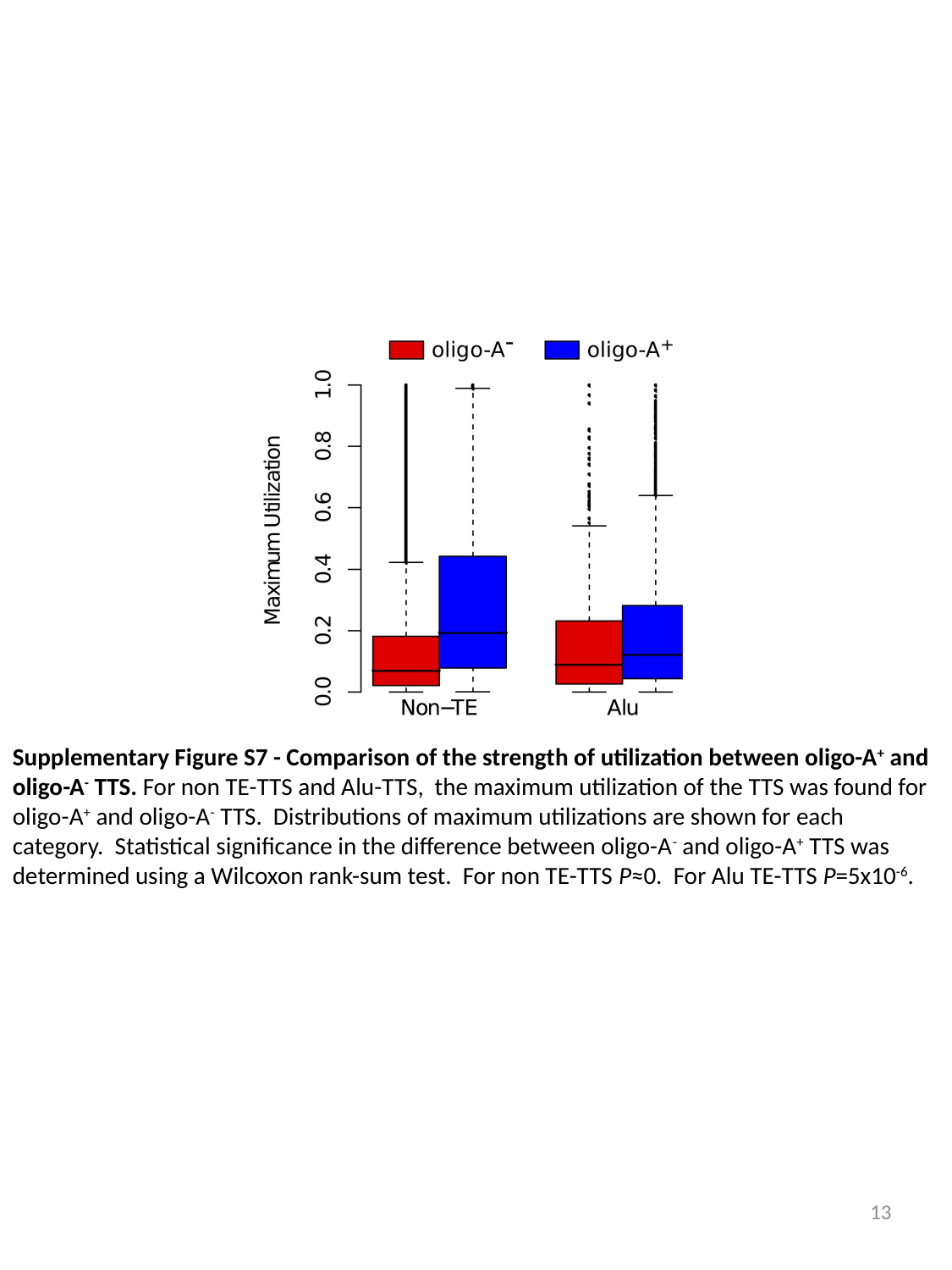

Supplementary Figure S7 - Comparison of the strength of utilization between oligo-A+ and oligo-A- TTS. For non TE-TTS and Alu-TTS, the maximum utilization of the TTS was found for oligo-A+ and oligo-A- TTS. Distributions of maximum utilizations are shown for each category. Statistical significance in the difference between oligo-A- and oligo-A+ TTS was determined using a Wilcoxon rank-sum test. For non TE-TTS P≈0. For Alu TE-TTS P=5x10^-6^.
13

## Slide 14
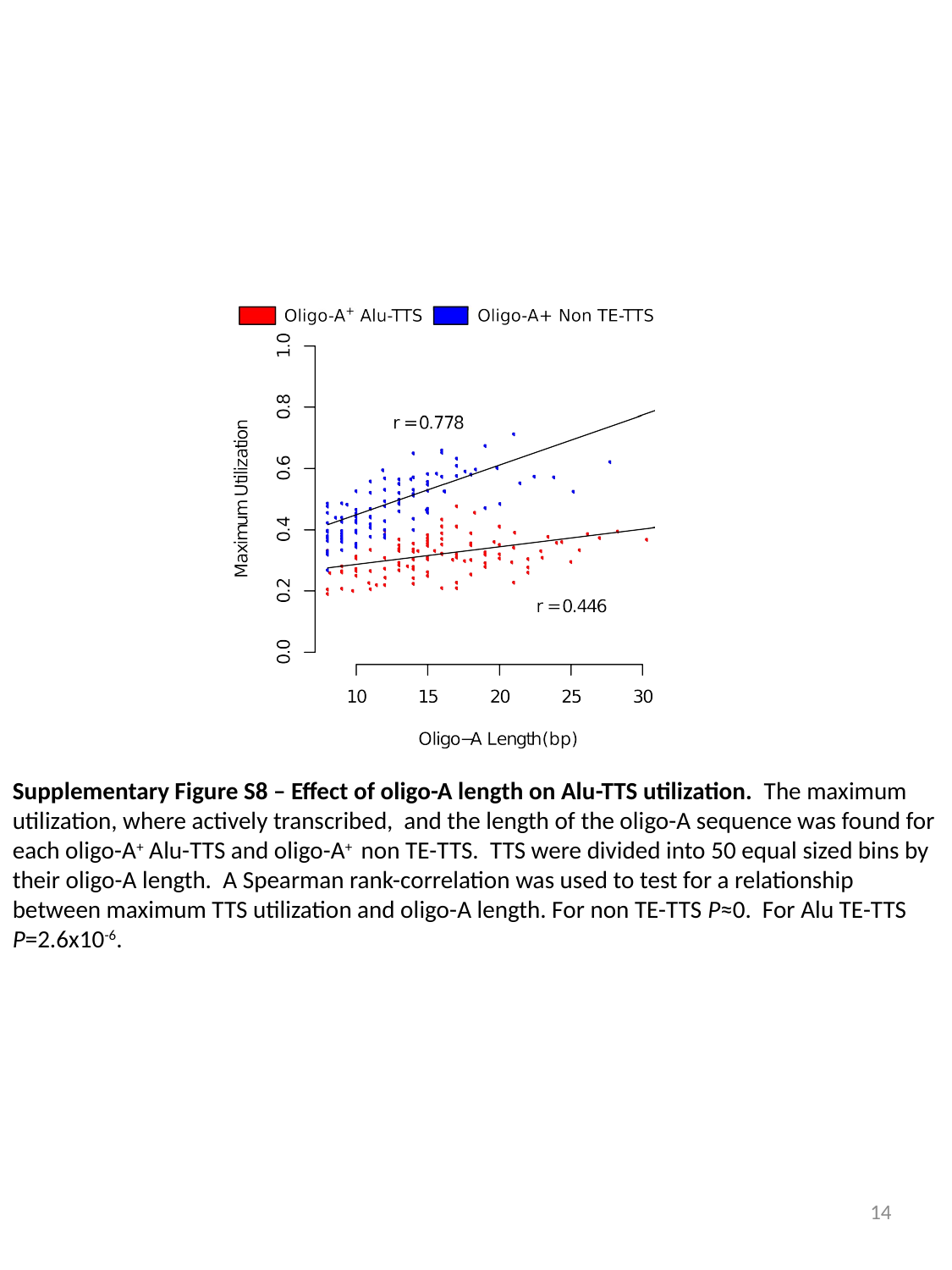

Supplementary Figure S8 – Effect of oligo-A length on Alu-TTS utilization. The maximum utilization, where actively transcribed, and the length of the oligo-A sequence was found for each oligo-A+ Alu-TTS and oligo-A+ non TE-TTS. TTS were divided into 50 equal sized bins by their oligo-A length. A Spearman rank-correlation was used to test for a relationship between maximum TTS utilization and oligo-A length. For non TE-TTS P≈0. For Alu TE-TTS P=2.6x10^-6^.
14

## Slide 15
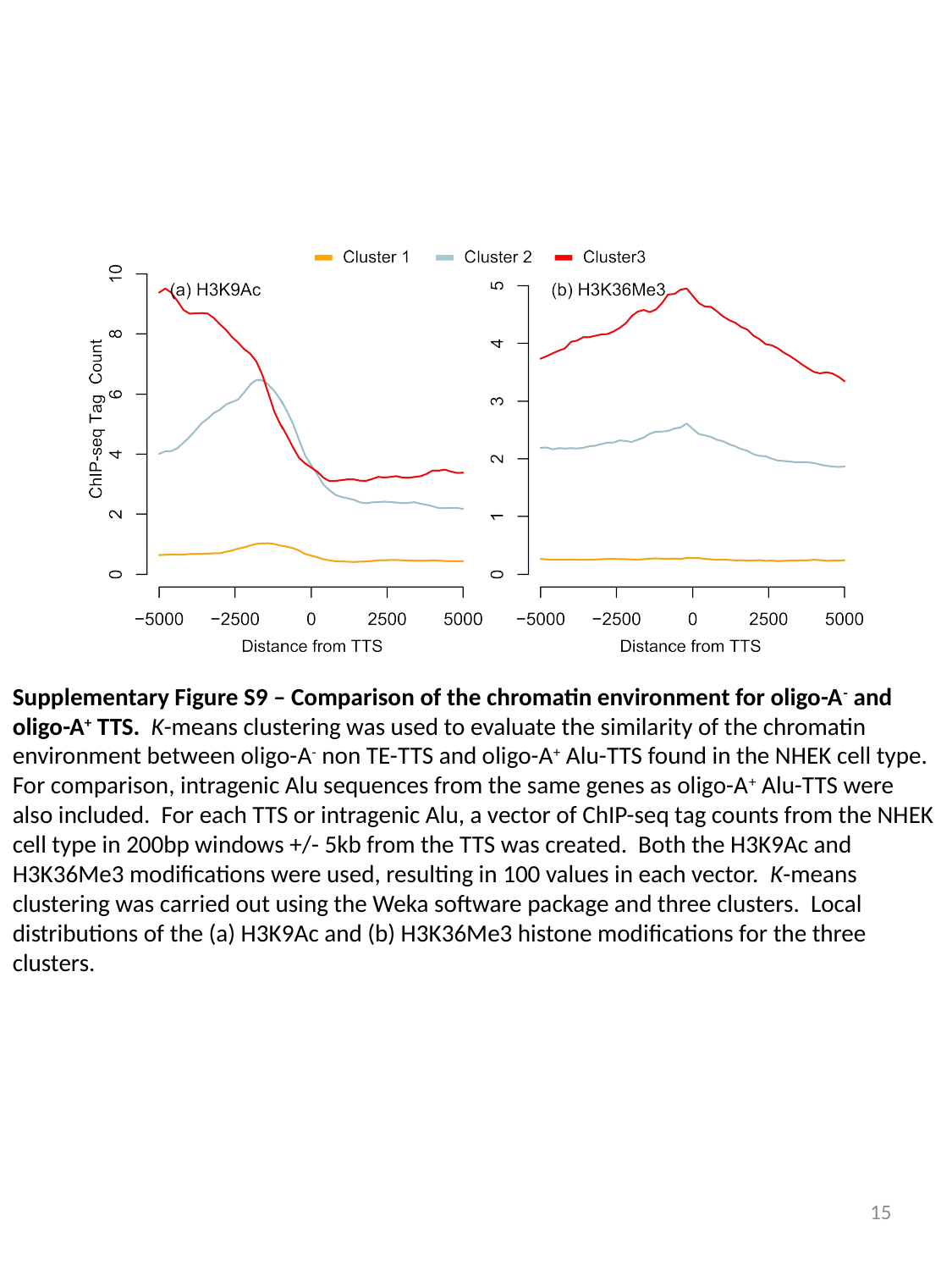

Supplementary Figure S9 – Comparison of the chromatin environment for oligo-A- and oligo-A+ TTS. K-means clustering was used to evaluate the similarity of the chromatin environment between oligo-A- non TE-TTS and oligo-A+ Alu-TTS found in the NHEK cell type. For comparison, intragenic Alu sequences from the same genes as oligo-A+ Alu-TTS were also included. For each TTS or intragenic Alu, a vector of ChIP-seq tag counts from the NHEK cell type in 200bp windows +/- 5kb from the TTS was created. Both the H3K9Ac and H3K36Me3 modifications were used, resulting in 100 values in each vector. K-means clustering was carried out using the Weka software package and three clusters. Local distributions of the (a) H3K9Ac and (b) H3K36Me3 histone modifications for the three clusters.
15

## Slide 16
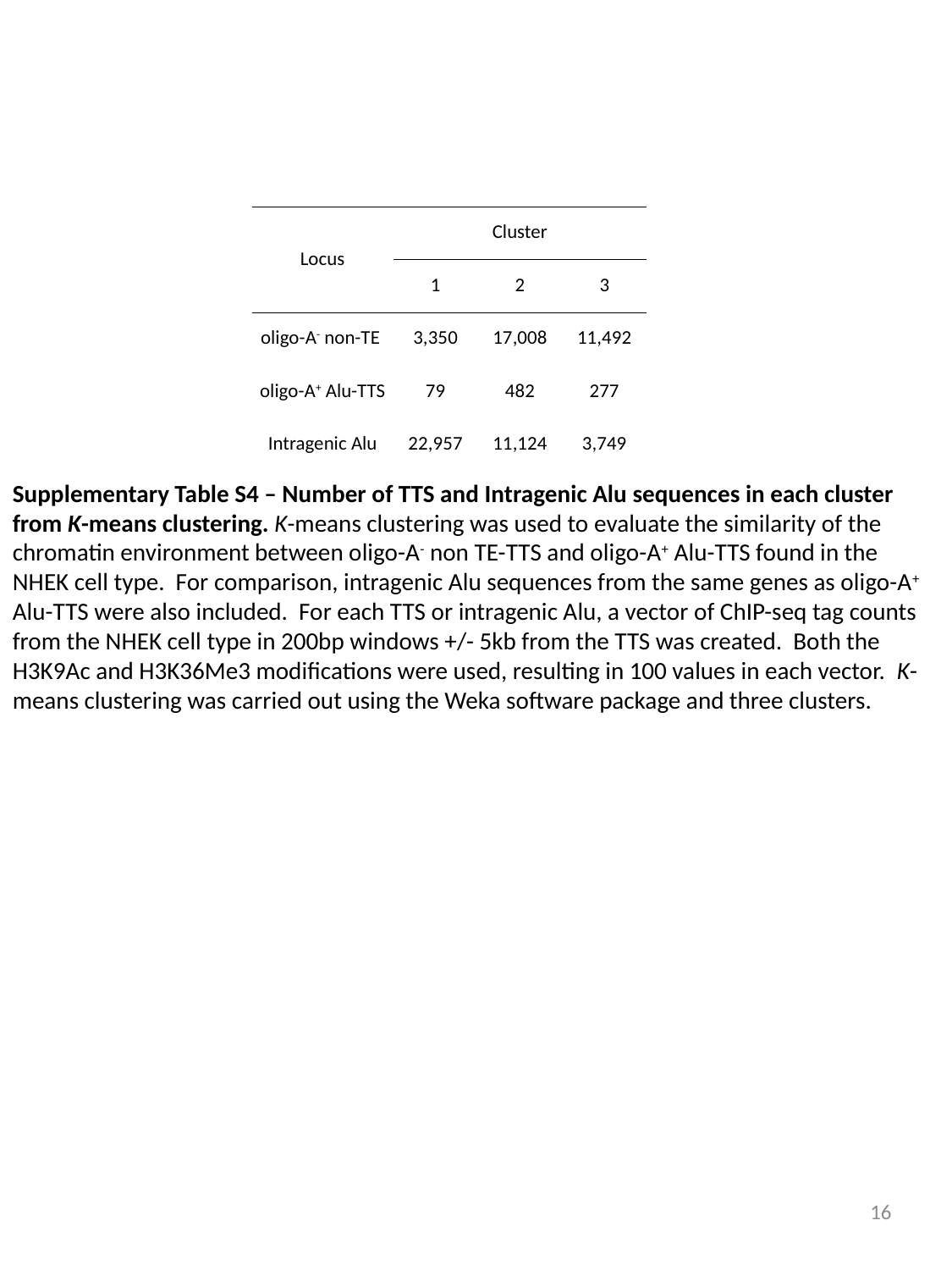

| Locus | Cluster | | |
| --- | --- | --- | --- |
| | 1 | 2 | 3 |
| oligo-A- non-TE | 3,350 | 17,008 | 11,492 |
| oligo-A+ Alu-TTS | 79 | 482 | 277 |
| Intragenic Alu | 22,957 | 11,124 | 3,749 |
Supplementary Table S4 – Number of TTS and Intragenic Alu sequences in each cluster from K-means clustering. K-means clustering was used to evaluate the similarity of the chromatin environment between oligo-A- non TE-TTS and oligo-A+ Alu-TTS found in the NHEK cell type. For comparison, intragenic Alu sequences from the same genes as oligo-A+ Alu-TTS were also included. For each TTS or intragenic Alu, a vector of ChIP-seq tag counts from the NHEK cell type in 200bp windows +/- 5kb from the TTS was created. Both the H3K9Ac and H3K36Me3 modifications were used, resulting in 100 values in each vector. K-means clustering was carried out using the Weka software package and three clusters.
16

## Slide 17
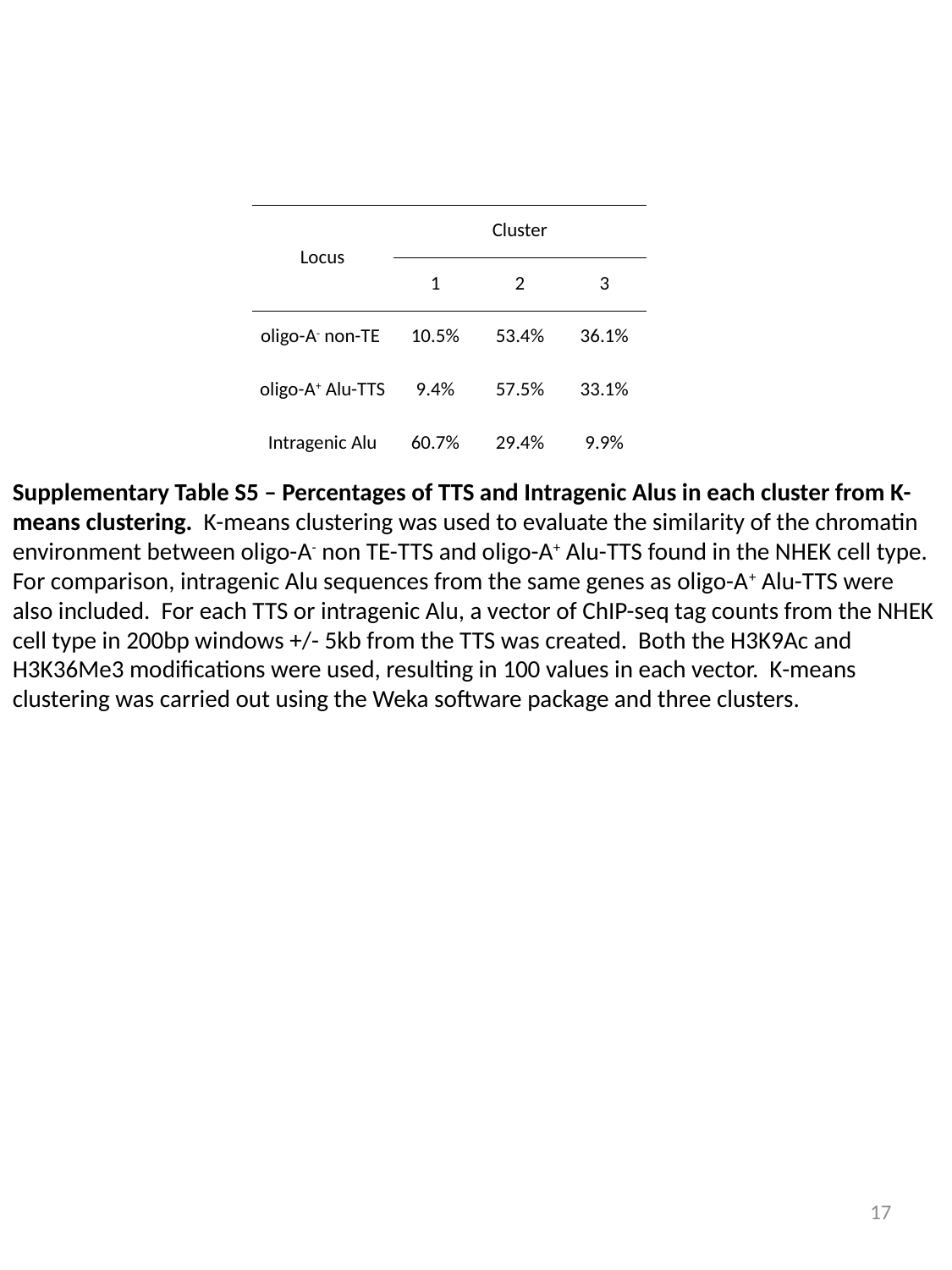

| Locus | Cluster | | |
| --- | --- | --- | --- |
| | 1 | 2 | 3 |
| oligo-A- non-TE | 10.5% | 53.4% | 36.1% |
| oligo-A+ Alu-TTS | 9.4% | 57.5% | 33.1% |
| Intragenic Alu | 60.7% | 29.4% | 9.9% |
Supplementary Table S5 – Percentages of TTS and Intragenic Alus in each cluster from K-means clustering. K-means clustering was used to evaluate the similarity of the chromatin environment between oligo-A- non TE-TTS and oligo-A+ Alu-TTS found in the NHEK cell type. For comparison, intragenic Alu sequences from the same genes as oligo-A+ Alu-TTS were also included. For each TTS or intragenic Alu, a vector of ChIP-seq tag counts from the NHEK cell type in 200bp windows +/- 5kb from the TTS was created. Both the H3K9Ac and H3K36Me3 modifications were used, resulting in 100 values in each vector. K-means clustering was carried out using the Weka software package and three clusters.
17
